# ECHDC2 inhibits the proliferation of gastric cancer cells by binding with NEDD4 to degrade MCCC2 and reduce aerobic glycolysis

**DOI:** 10.1186/s10020-024-00832-9

**Published:** 2024-05-23

**Authors:** Jiancheng He, Jianfeng Yi, Li Ji, Lingchen Dai, Yu Chen, Wanjiang Xue

**Affiliations:** 1 Department of Gastrointestinal Surgery, Affliated Hospital of Nantong University, Medical School of Nantong University, 20 Xisi Street, Nantong, 226001 China; 2 Research Center of Clinical Medicine, Affiliated Hospital of Nantong University, Nantong, 226001 China; 3 Nantong Key Laboratory of Gastrointestinal Oncology, Nantong, 226001 China

**Keywords:** Gastric cancer, ECHDC2, Aerobic glycolysis, MCCC2, NEDD4, Ubiquitination

## Abstract

**Background:**

The Enoyl-CoA hydratase/isomerase family plays a crucial role in the metabolism of tumors, being crucial for maintaining the energy balance and biosynthetic needs of cancer cells. However, the enzymes within this family that are pivotal in gastric cancer (GC) remain unclear.

**Methods:**

We employed bioinformatics techniques to identify key Enoyl-CoA hydratase/isomerase in GC. The expression of ECHDC2 and its clinical significance were validated through tissue microarray analysis. The role of ECHDC2 in GC was further assessed using colony formation assays, CCK8 assay, EDU assay, Glucose and lactic acid assay, and subcutaneous tumor experiments in nude mice. The mechanism of action of ECHDC2 was validated through Western blotting, Co-immunoprecipitation, and immunofluorescence experiments.

**Results:**

Our analysis of multiple datasets indicates that low expression of *ECHDC2* in GC is significantly associated with poor prognosis. Overexpression of *ECHDC2* notably inhibits aerobic glycolysis and proliferation of GC cells both in vivo and in vitro. Further experiments revealed that overexpression of *ECHDC2* suppresses the P38 MAPK pathway by inhibiting the protein level of MCCC2, thereby restraining glycolysis and proliferation in GC cells. Ultimately, it was discovered that ECHDC2 promotes the ubiquitination and subsequent degradation of MCCC2 protein by binding with NEDD4.

**Conclusions:**

These findings underscore the pivotal role of the ECHDC2 in regulating aerobic glycolysis and proliferation in GC cells, suggesting ECHDC2 as a potential therapeutic target in GC.

**Supplementary Information:**

The online version contains supplementary material available at 10.1186/s10020-024-00832-9.

## Background

Gastric Cancer (GC) as the fifth most common malignant tumor globally, characterized by high incidence and mortality rates. Owing to the subtle initial symptoms of GC, the majority of patients are diagnosed at advanced stages, which results in a poor prognosis (Smyth et al. 2020). Currently, surgery and chemoradiotherapy are the primary treatment modalities (Parisi et al. 2020). Despite advancements in diagnostic and treatment technologies enhancing therapeutic outcomes, the overall prognosis for GC remains poor, with common postoperative recurrences and drug resistance (Baccili Cury Megid et al. 2023). Therefore, a deeper exploration of the molecular mechanisms of GC cells and the development of targeted treatment strategies are crucial for improving treatment efficacy.

GC undergoes profound metabolic reprogramming during its development, with glycolysis playing a pivotal role in supporting its aggressive growth and spread (Zhao et al. 2022; Tan et al. 2022; Xu et al. 2023). This intricate metabolic adjustment not only provides GC with the essential energy and biosynthetic precursors needed for rapid proliferation but also alters the surrounding microenvironment to facilitate cancer progression, thereby highlighting the complexity and high adaptability of GC’s metabolic strategies (Sun et al. 2023; Li and Ma 2021; Kadam et al. 2021). Aerobic glycolysis, also known as the Warburg effect, allows cancer cells to produce a significant amount of lactate even in the presence of ample oxygen, supplying the tumor with energy and compounds that support growth (DeBerardinis and Chandel 2020; Liberti and Locasale 2016). In this process, key enzymes such as Pyruvate kinase M2 (PKM2) and glucose transporter type 1 (GLUT1) play a crucial role (Li et al. 2018; Iacobini et al. 2023; Yu et al. 2023; Wu et al. 2023). Targeting the genes involved in the aerobic glycolysis pathway becomes essential for inhibiting GC cell growth and promoting personalized treatment strategies that leverage the unique metabolic profile of GC, enhancing therapeutic efficacy while minimizing side effects (Tao et al. 2023; Wang et al. 2022; Cui et al. 2022). However, despite the significance of these strategies, the mechanisms underlying aerobic glycolysis in GC are not fully elucidated, underscoring the need for further investigation into the critical mechanisms regulating glycolysis.

Existing research has demonstrated that alterations in fatty acid β-oxidation can impact glycolysis. Schlaepfer et al. discovered that in prostate cancer mouse xenografts, reducing fatty acid β-oxidation significantly impacts glycolysis (Schlaepfer et al. 2015). Yuying Tan et al. discovered a metabolic reprogramming in cisplatin-resistant cancer cells, shifting from glycolysis to fatty acid uptake and β-oxidation (Tan et al. 2022).Fatty acid β-oxidation is a crucial metabolic process occurring in the mitochondria, where fatty acids are broken down into acetyl-CoA units, generating ATP, NADH, and FADH2, which fuel cellular energy needs (Gomez-Gutierrez et al. 2015). Zhen Xiong et al. confirmed fatty acid β-oxidation and other lipid peroxidation processes were reduced in GC (Xiong et al. 2021). The enoyl-CoA hydratase/isomerase family is critical to fatty acid metabolism, facilitating energy production and cellular functions through β-oxidation. Their involvement in tumor metabolism connects key metabolic pathways, such as fatty acid β-oxidation and aerobic glycolysis, supporting cancer cell growth and adaptation (Muller-Newen et al. 1995; Padavattan et al. 2021; Hwang et al. 2020; Agnihotri and Liu 2003). Enoyl-CoA hydratase 1 (ECH1), as a vital component of mitochondrial fatty acid β-oxidation, can improve various pathological states in mouse liver upon overexpression (Liu et al. 2021a, b). Enoyl-CoA hydratase and 3-hydroxyacyl CoA dehydrogenase (EHHADH) influences lipid metabolism and aerobic glycolysis in ovarian cancer (Zhao et al. 2010; Lee et al. 2022). Hydroxyacyl-CoA dehydrogenase trifunctional multienzyme complex subunit alpha (HADHA), a mitochondrial enzyme, catalyzes the β-oxidation of long-chain fatty acids and is also associated with cellular energy metabolism and diabetes (Liu et al. 2020a, b; Pan et al. 2022). However, the key enoyl-CoA hydratase/isomerases and their mechanisms of action in GC remain unclear.

In this study, we demonstrate for the first time that Enoyl-CoA Hydratase Domain Containing 2 (ECHDC2) is downregulated in GC, correlating with poor prognosis. The findings indicate that ECHDC2 can inhibit the proliferation and aerobic glycolysis of GC cells. The mechanism primarily involves its regulation of E3 ubiquitin ligase NEDD4-mediated ubiquitination and degradation of Methylcrotonyl-CoA Carboxylase Subunit 2 (MCCC2), which subsequently suppresses the expression of the *PKM2* and *GLUT1*.

## Results

### ECHDC2 is downregulated in GC tissues

**Fig. 1:**
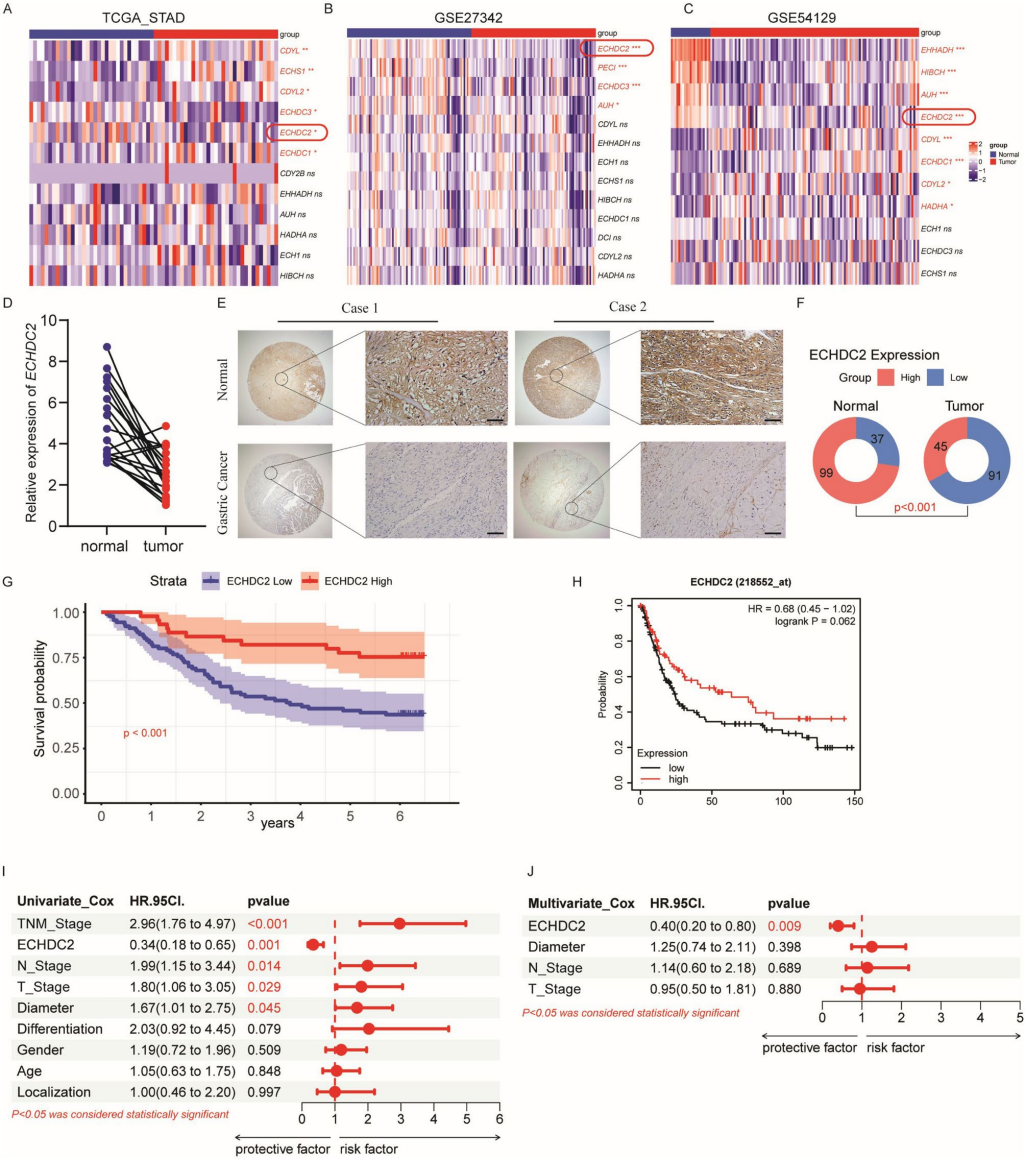
ECHDC2 is downregulated in GC tissues. (**A-C**) Heatmaps depict the differential expression of various enoyl-CoA hydratase/isomerases in GC versus normal tissues in the TCGA-STAD, GSE27342, and GSE54129 datasets. (**D**) Relative expression levels of ECHDC2 in 20 GC tissues and their corresponding adjacent non-cancerous tissues. (**E**) Representative images of tissue microarrays. Scale bar, 50 μm. (**F**) Quantitative analysis of ECHDC2 protein expression. (**G**) Kaplan-Meier survival curve analysis explores the relationship between ECHDC2 expression levels in tissue microarrays and overall survival (OS) in patients with GC. (**H**) Analysis of the relationship between ECHDC2 expression and GC patient OS based on the Kaplan-Meier Plotter database. (**I–J**) Univariate and multivariate Cox regression analyses of GC patients in tissue microarrays. * *P* < 0.05, ** *P* < 0.01, *** *P* < 0.001

To investigate the key enzyme in the Enoyl-CoA hydratase/isomerase family implicated in GC, we analyzed three datasets: TCGA-STAD, GSE27342, and GSE54129. The findings highlight *ECHDC2* as the uniquely significant gene consistently identified across three databases for being notably downregulated in GC tissues compared to normal tissues (Fig. 1A-C, Supplementary Figure S1A-C). To validate this discovery, we conducted qRT-PCR assays and western blotting assay on 20 pairs of fresh GC tissues and their corresponding adjacent tissues. The results were consistent with those from the datasets, indicating a low expression of ECHDC2 in GC (Fig. 1D, Supplementary Figure S1D). Subsequently, using IHC, we analyzed the relative expression levels of ECHDC2 protein in 136 GC cases and their adjacent non-cancerous tissues within the TMA. Our findings indicated a significant relative decrease in ECHDC2 expression in the GC tissues compared to the non-cancerous counterparts (Fig. 1E-F). Next, we analyzed the correlation between the expression of ECHDC2 and various clinicopathological characteristics in a cohort of 136 patients. The results revealed that low expression of ECHDC2 was positively correlated with the depth of invasion, lymph node metastasis and TNM stage, but it was not associated with gender, age, degree of differentiation, tumor diameter, tumor localization (Table 1). Kaplan-Meier survival curve analysis revealed that GC patients with low ECHDC2 expression have a poor prognosis (Fig. 1G). This finding was also validated in The Kaplan-Meier Plotter database (Fig. 1H). Univariate and multivariate Cox regression analyses further confirmed that ECHDC2 is an independent predictor factor in GC (Fig. 1I-J). In conclusion, the low expression of ECHDC2 in GC is a key factor in the progression of the disease.

**Table 1:** Correlation between ECHDC2 expression in GC tissue and clinicopathological Features of GC patients

Clinicopathological parameter	Total (*n* = 136)	ECHDC2 expression		*p* value
		Low(*n* = 91)	High(*n* = 45)	
Age(years)				0.220
≤ 65	55	33	22	
> 65	81	59	23	
Gender				0.447
Male	53	38	15	
Female	83	53	30	
Lymph node metastasis				0.036
Negative(N0)	54	30	24	
Positive(N1-N3)	82	61	21	
TNM stage				< 0.001
I	36	15	21	
II	69	52	17	
III	31	24	7	
Depth of invasion				< 0.001
T1	24	8	16	
T2	35	22	13	
T3	65	52	13	
T4	12	9	3	
Tumor diameter (cm)				0.522
< 5	84	54	30	
≥ 5	52	37	15	
Tumor differentiation				0.456
Well	24	14	10	
Moderate/Poor	112	77	35	
Tumor location				0.255
Up	15	8	7	
Middle/Down	121	83	38	

### ECHDC2 inhibits the proliferation of GC cells in vivo and in vitro

**Fig. 2:**
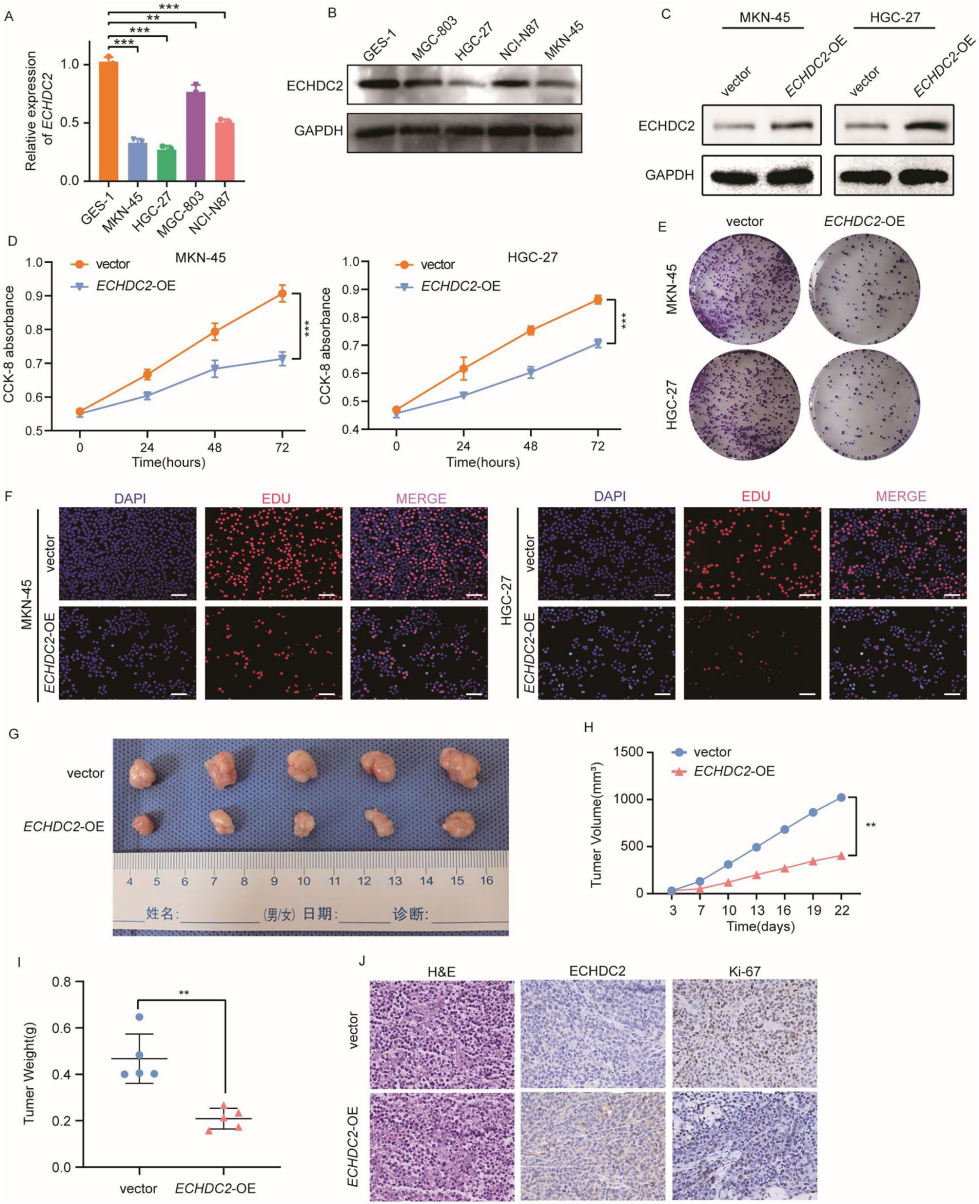
ECHDC2 inhibits GC cell proliferation in vivo and in vitro. (**A**) qRT-PCR was used to detect the relative mRNA expression levels of ECHDC2 in GES-1 and four GC cell lines. (**B**) Western blotting was performed to detect the protein expression of ECHDC2 in GES-1 and four GC cell lines. (**C**) Western blotting was used to detect transfection efficiency after overexpression of ECHDC2. (**D**) GC cells was detected by CCK-8 assay to cell viability. (**E–F**) Colony formation assay and EDU assay were performed to assess the cell proliferation capacity. Scale bar, 20 μm. (**G**) Typical images of subcutaneous xenograft tumor. (**H–I**) The volume and weight of subcutaneous xenograft tumors (*n* = 5). (**J**) Intensity of IHC staining in different groups of subcutaneous xenograft tumor. Scale bar, 50 μm. * *P* < 0.05, ** *P* < 0.01, *** *P* < 0.001

To explore the role of ECHDC2 in GC cells, we examined the expression of *ECHDC2* in four GC cell lines MKN-45, HGC-27, MGC-803 and NCI-N87, as well as in a normal gastric mucosal cell line GES-1. qRT-PCR and western blotting assays demonstrated that the expression of ECHDC2 in GC cell lines was lower than in GES-1, with the lowest expression observed in HGC-27 and MKN-45 (Fig. 2A, B). Subsequently, we constructed *ECHDC2* stably overexpressing cell lines using MKN-45 and HGC-27 cells. Western blotting demonstrated significant overexpression of ECHDC2 (Fig. 2C). CCK-8 assay, clonogenic assay, and Edu assay results indicated that the proliferation ability of GC cells in the *ECHDC2* overexpression group was significantly lower than that of the control group (Fig. 2D-F). In order to further verify whether *ECHDC2* overexpression could inhibit the proliferation of GC cells in vivo, we constructed a subcutaneous tumor model in nude mice. The results showed the tumor sizes and weights of *ECHDC2* overexpression group were found to be significantly lower than those of the control group (Fig. 2G-I). IHC staining of subcutaneous xenograft tumors in nude mice showed that compared to the control group, the group with overexpression of *ECHDC2* exhibited lower expression of Ki-67 (Fig. 2J). The above experimental results demonstrated that *ECHDC2* overexpression inhibited the growth of GC cells in vivo and in vitro.

### ECHDC2 inhibits aerobic glycolysis in GC cells

**Fig. 3:**
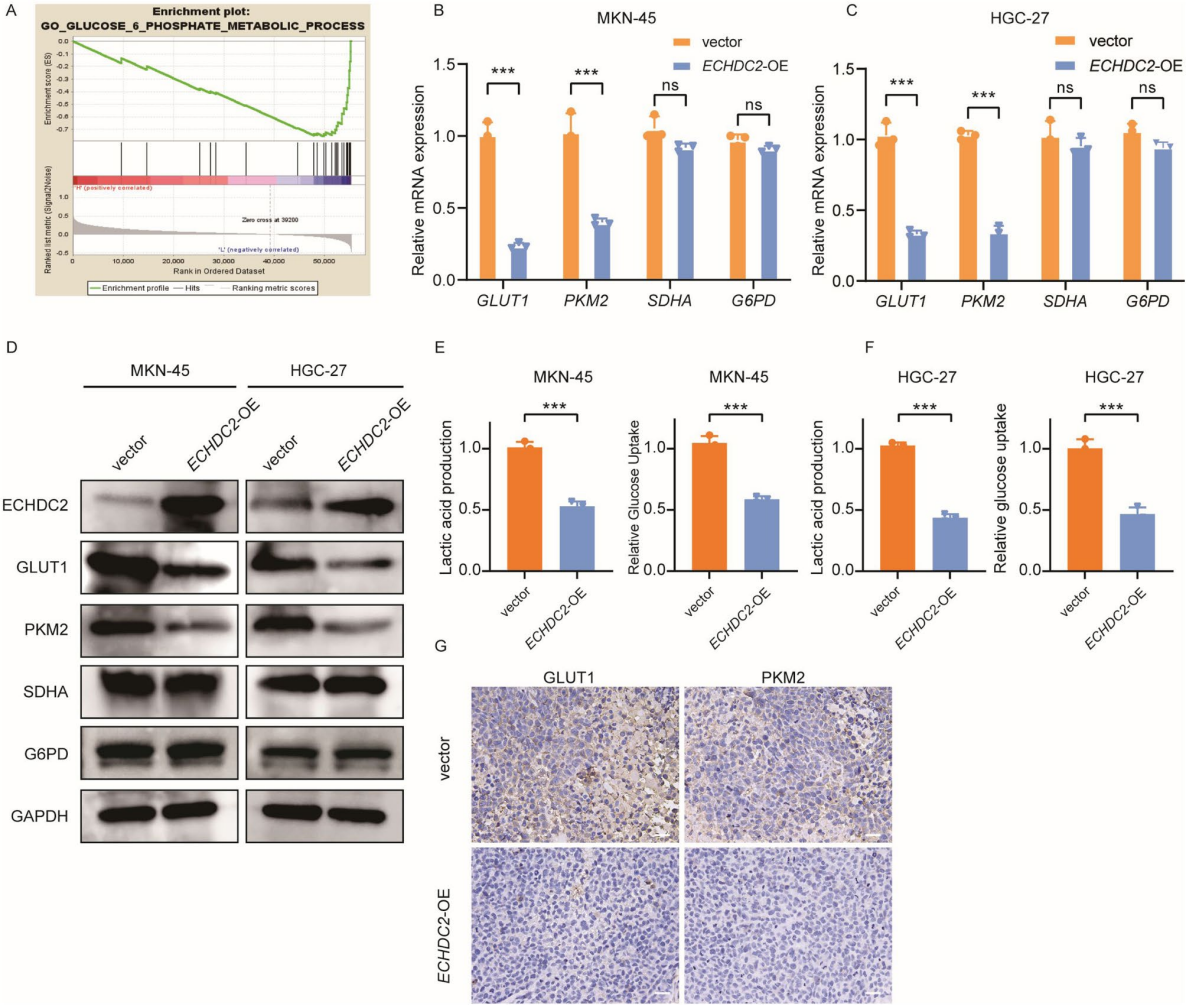
ECHDC2 inhibits aerobic glycolysis in GC cells. (**A**) GSEA results of the TCGA-STAD dataset show that ECHDC2 is associated with glycolysis. (**B–C**) qRT-PCR was used to detect the mRNA expression of GLUT1, PKM2, SDHA and G6PD after overexpression of ECHDC2. (**D**) Effect of ECHDC2 expression on proteins related to aerobic oxidation, aerobic glycolysis, and pentose phosphate pathway detected by western blotting. (**E–F**) Glucose uptake rate and lactic acid production rate of GC cells were measured after ECHDC2 overexpression. (**G**) IHC analysis of subcutaneous xenograft tumor was performed with anti-GLUT1 antibody and anti-PKM2 antibody. Scale bar, 50 μm. ns *P* > 0.05, ** *P* < 0.01, *** *P* < 0.001

To further explore the mechanism by which ECHDC2 affects the proliferative ability of GC cells, we utilized the TCGA-STAD dataset to divide GC patients into two groups of high and low expression based on the median *ECHDC2* expression. The biological processes regulated by *ECHDC2* were explored by gene set enrichment analysis (GSEA), and the results suggested that ECHDC2 was associated with the glucose metabolism pathway (Fig. 3A). Sugar metabolism is mainly divided into three ways: aerobic oxidation, aerobic glycolysis, and pentose phosphate pathway(Zhang et al. 2023; Wei et al. 2023; TeSlaa et al. 2023). In order to clarify the pathways through which ECHDC2 affects the proliferation of GC cells, we detected the intermediate metabolites of the three pathways, qRT-PCR and western blotting revealed that, compared to the control group, the expression of key glycolytic enzymes PKM2 and GLUT1 was significantly reduced in the *ECHDC2* overexpression group, while there was no significant difference in the expression of key aerobic oxidation enzyme Succinate Dehydrogenase (SDH) and pentose phosphate pathway enzyme Glucose-6-Phosphate Dehydrogenase (G6PD) (Rustin et al. 2002; Zhong et al. 2021) (Fig. 3B-D). To verify whether ECHDC2 is involved in regulating the aerobic glycolysis process in GC cells, we further examined the effects of overexpression of *ECHDC2* on the lactic acid production rate and glucose uptake rate in GC cells. The results showed that the rate of glucose uptake and lactate production in GC cells of the *ECHDC2* overexpression group was significantly lower than that of the control group (Fig. 3E-F). IHC staining of subcutaneous xenograft tumors in nude mice showed that compared to the control group, the group with overexpression of *ECHDC2* exhibited lower expression of PKM2, and GLUT1 (Fig. 3G). The above results suggest that overexpression of *ECHDC2* can significantly inhibit aerobic glycolysis in GC cells.

### ECHDC2 inhibits aerobic glycolysis and proliferation in GC cells via the P38 MAPK pathway

**Fig. 4:**
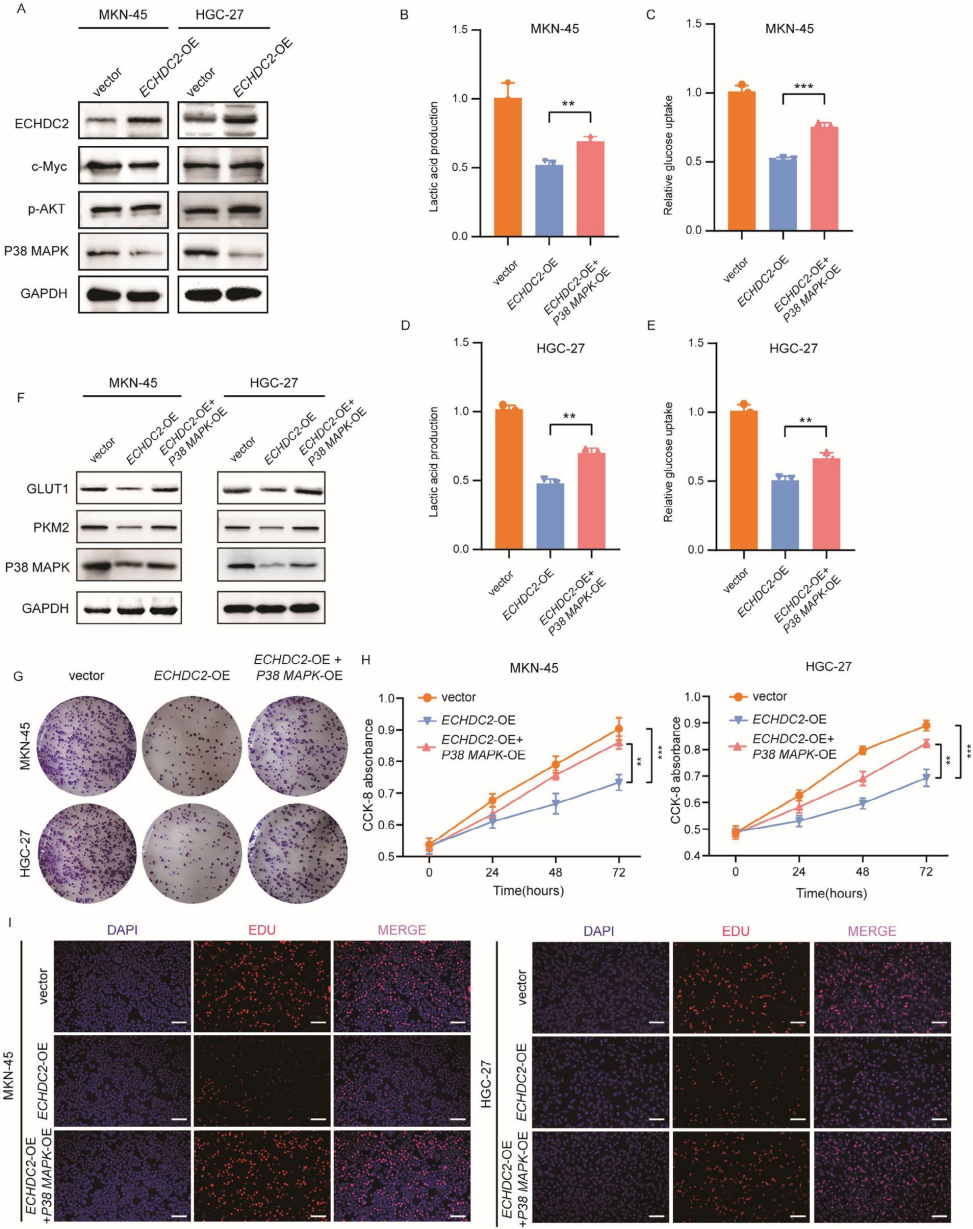
ECHDC2 inhibits aerobic glycolysis and cell proliferation via the P38 MAPK pathway. (**A**) Western blotting was used to detect the effect of ECHDC2 on PI3K-AKT/mTOR pathway, Myc pathway, and P38-MAPK pathway. (**B-E**) Glucose uptake rate and lactic acid production rate of GC cells were measured after ECHDC2 overexpression and/or P38 MAPK overexpression. (**F**) The protein expression of GLUT1, PKM2, P38 MAPK were detected in GC cells transfected with vector, ECHDC2 or ECHDC2 plus P38 MAPK by western blotting. (**G-I**) CCK-8 assay, colony formation assay and EDU assay were performed to assess the cell viability and cell proliferation capacity following ECHDC2 overexpression and/or P38 MAPK overexpression. Scale bar, 20 μm. **P < 0.01, *** *P* < 0.001

To explore the mechanism by which ECHDC2 regulates aerobic glycolysis in GC cells, we analyzed several common signaling pathways that affect aerobic glycolysis, including the PI3K-AKT/mTOR pathway, the Myc pathway, and the P38 MAPK pathway (DiToro et al. 2020; Wang et al. 2019, 2021; Liu et al. 2021a, b). Western blotting results showed that the expression of P38 MAPK in the *ECHDC2* overexpression group was significantly lower than in the control group, while the expression of p-AKT and c-Myc showed no difference (Fig. 4A). Existing research indicates that the P38 MAPK signaling pathway can regulate the expression of *PKM2* and *GLUT1*. To investigate whether ECHDC2 influences GC cell aerobic glycolysis and proliferation by regulating PKM2 and GLUT1 via the P38 MAPK pathway, we overexpressed *P38 MAPK* in *ECHDC2* overexpressing cell lines. We found that the inhibition of aerobic glycolysis and proliferation in GC cells by the sole overexpression of *ECHDC2* was restored in the group with simultaneous overexpression of *ECHDC2* and *P38 MAPK* (Fig. 4B-I). The results suggest that ECHDC2 inhibits aerobic glycolysis and proliferation of GC cells through the P38 MAPK signaling pathway.

### ECHDC2 inhibits aerobic glycolysis and proliferation in GC cells by promoting the ubiquitination and degradation of MCCC2

**Fig. 5:**
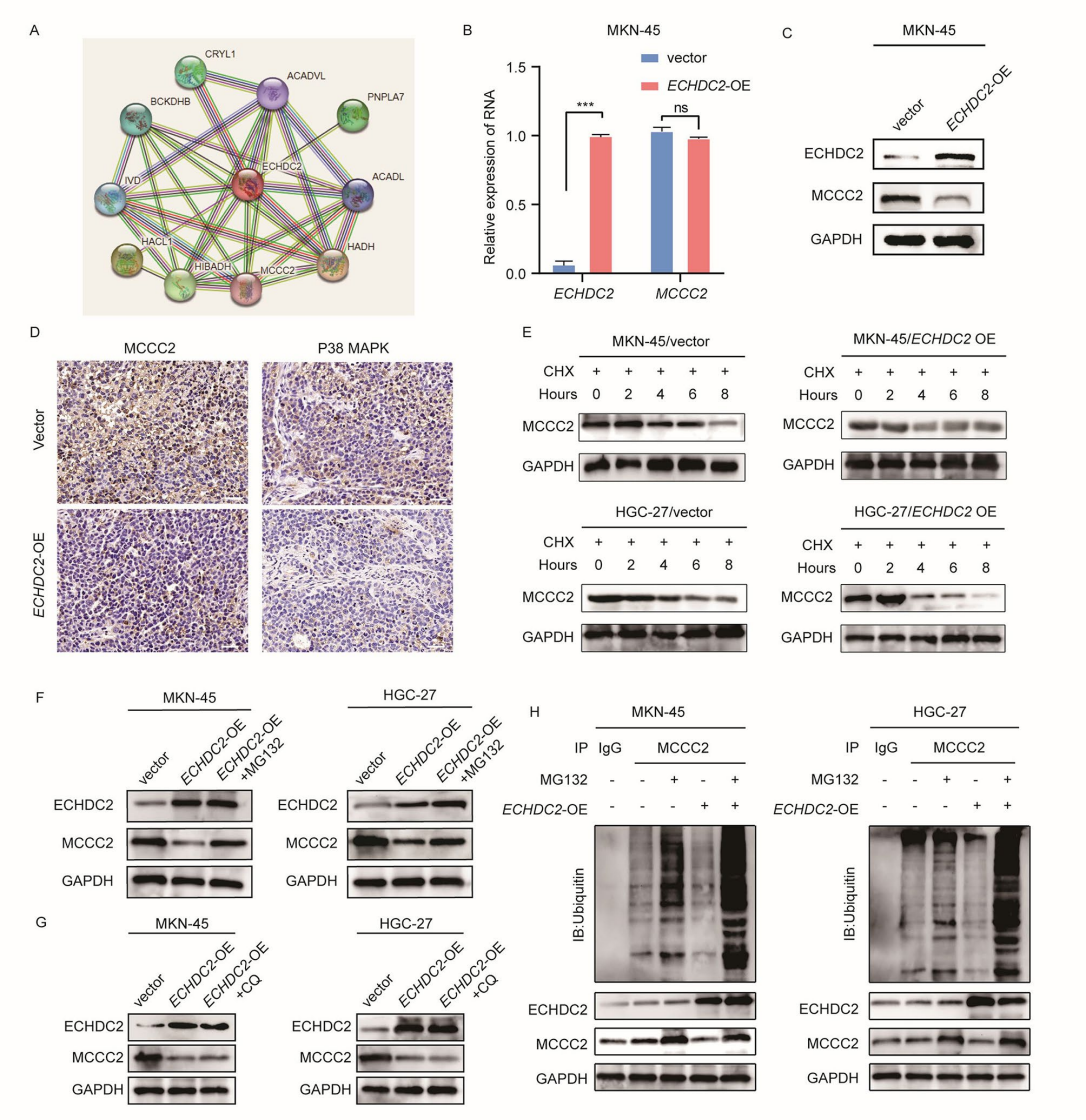
ECHDC2 promotes the degradation of MCCC2 through the ubiquitin-proteasome pathway. (**A**) STRING predicts the interacting proteins of ECHDC2. (**B–C**) qRT-PCR and western blotting were employed to detect the mRNA and protein expression of ECHDC2 and MCCC2 after overexpression of ECHDC2. (**D**) IHC was performed to detect the protein expression of MCCC2 and P38 MAPK in subcutaneous xenograft tumor. Scale bar, 50 μm. (**E**) Western blotting was used to detect the effect of ECHDC2 overexpression on MCCC2 protein stability after treatment with CHX. (**F–G**) MCCC2 protein levels were detected in ECHDC2 overexpressing GC cells treated with MG132 or CQ. (**H**) The effect of ECHDC2 on the ubiquitination level of MCCC2 was detected in GC cells treated with/without MG132. ns *P* > 0.05, *** *P* < 0.001

To investigate how ECHDC2 affects the P38 MAPK pathway to regulate aerobic glycolysis and proliferation in GC cells, we utilized the STRING database to analyze the proteins associated with ECHDC2 (Fig. 5A). Previous research has shown that MCCC2 can activate the P38 MAPK pathway to enhance aerobic glycolysis in prostate cancer (He et al. 2020). To verify whether MCCC2 is a downstream target of ECHDC2, we first examined if ECHDC2 could affect the expression of MCCC2. qRT-PCR and Western blotting results suggested that the protein level of MCCC2 in the *ECHDC2* overexpression group was significantly lower than in the control group, while there was no difference at the mRNA level (Fig. 5B-C). IHC staining of subcutaneous xenograft tumors in nude mice showed that compared to the control group, the group with overexpression of *ECHDC2* exhibited lower expression of MCCC2 and P38 MAPK (Fig. 5D). To further investigate the impact of ECHDC2 on the protein levels of MCCC2, we first treated cells with CHX. The results suggested that in the *ECHDC2* overexpression group, the degradation rate of MCCC2 protein was significantly faster compared to the control group (Fig. 5E). The ubiquitin-proteasome pathway and the autophagy-lysosome pathway are the two most important protein degradation pathways (Raffeiner et al. 2023; Cohen-Kaplan et al. 2016). To further investigate how ECHDC2 leads to the degradation of MCCC2 protein, we overexpressed *ECHDC2* and concurrently treated the cells with or without the proteasome inhibitor MG132 and the autophagy inhibitor CQ. We found that MG132 could inhibit the downregulation of MCCC2 protein levels in the *ECHDC2* overexpression group, while CQ could not (Fig. 5F-G). Further investigation revealed that the ubiquitination level in the *ECHDC2* overexpression group was significantly higher than that in the control group (Fig. 5H). Subsequently, we overexpressed *MCCC2* in *ECHDC2* overexpressing cell lines. Compared to the group with only *ECHDC2* overexpression, the aerobic glycolysis and proliferation capabilities of GC cells in the group with simultaneous overexpression of *ECHDC2* and *MCCC2* were restored (Supplementary Figure S2A-H).

### ECHDC2 mediates the ubiquitination and degradation of MCCC2 through NEDD4

**Fig. 6:**
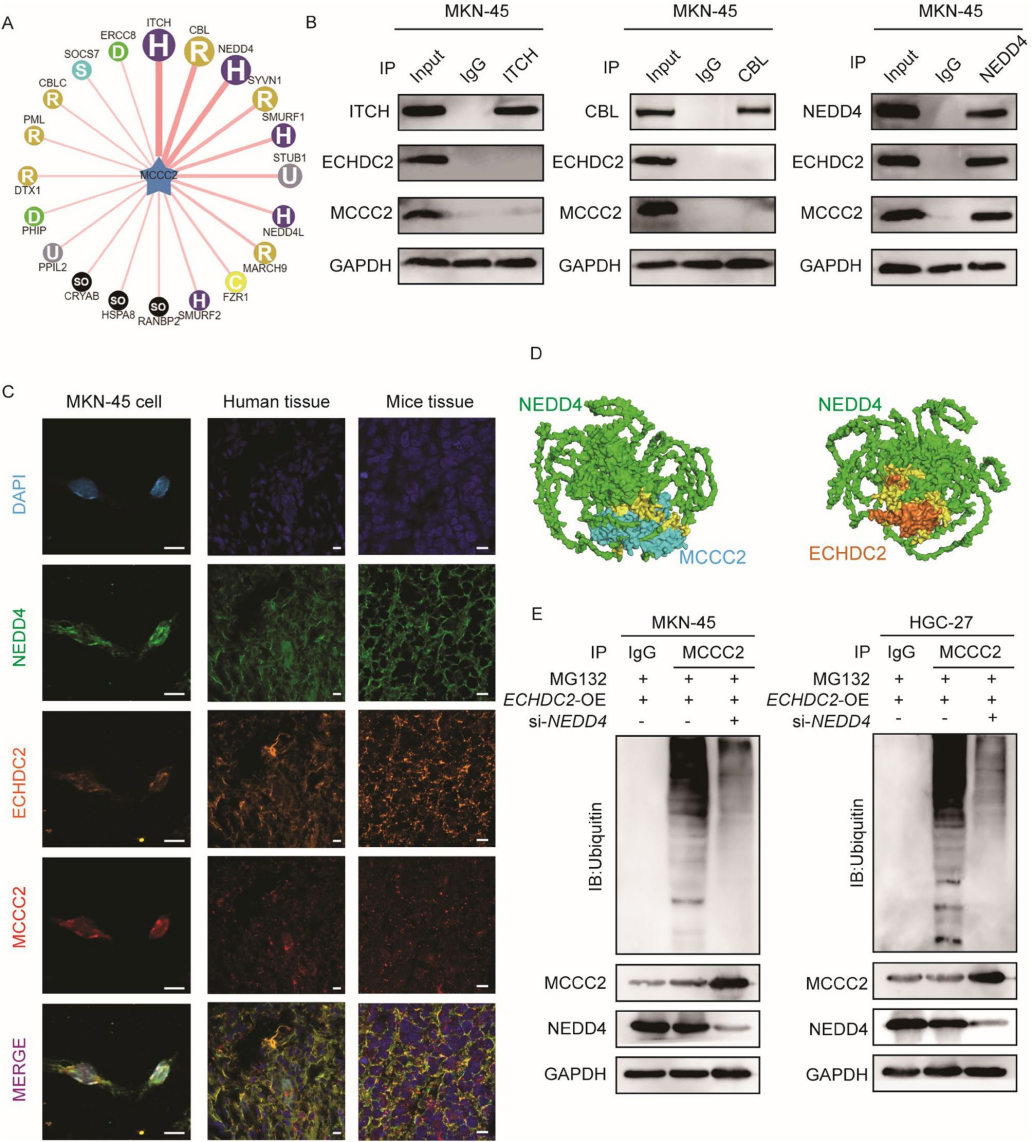
ECHDC2 ubiquitinates MCCC2 via NEDD4. (**A**) UbiBrowser was used to predict the E3 ubiquitin ligase of MCCC2. (**B**) Co-IP were used to validate the binding between ECHDC2, MCCC2 and NEDD4. (**C**) IF assays was used to detect co-localization of ECHDC2, MCCC2 and NEDD4 in GC cells, GC tissues and subcutaneous xenograft tumors. Scale bar, 10 μm. (**D**) Molecular docking models of ECHDC2 with NEDD4 and NEDD4 with MCCC2. Green: NEDD4; cyan: MCCC2; Orange: ECHDC2; Yellow: the docking region. (**E**) The effect of NEDD4 on ECHDC2-induced ubiquitination of MCCC2 was detected in GC cells

To further investigate the specific mechanism by which ECHDC2 promotes the ubiquitination of MCCC2, we initially used the UbiBrowser 1.0 database to predict potential E3 ubiquitin ligases for MCCC2. The top three predicted were ITCH, CBL, and NEDD4 (Fig. 6A). Subsequently, we conducted Co-IP experiments, which indicated that only NEDD4 interacts with both ECHDC2 and MCCC2 (Fig. 6B). This finding was corroborated by IF and mIHC, which also confirmed the colocalization of ECHDC2, NEDD4, and MCCC2 (Fig. 6C). Next, we engaged in protein-protein docking to anticipate the potential interaction patterns between ECHDC2 and NEDD4, and between NEDD4 and MCCC2 (Fig. 6D). To further validate whether the ECHDC2-promoted ubiquitination and degradation of MCCC2 is related to NEDD4, we knocked down NEDD4 while overexpressing ECHDC2. The results indicate that the ubiquitination of MCCC2, promoted by overexpression of ECHDC2, can be reversed by knocking down NEDD4 (Fig. 6E). The results indicate that ECHDC2 facilitates the ubiquitination and degradation of MCCC2 by binding to NEDD4.

## Discussion

In our study, we initially discovered that overexpression of *ECHDC2* can inhibit glycolysis and proliferation in GC cells. Further investigation into the underlying mechanisms revealed that ECHDC2 interacts with the E3 ubiquitin ligase NEDD4, promoting the ubiquitination and subsequent degradation of MCCC2. This effectively suppresses the activity of the P38 MAPK signaling pathway. The inhibition of the P38 MAPK pathway directly affects the expression levels of the key glycolytic enzymes PKM2 and GLUT1, thereby further hindering the glycolytic process and the proliferative capacity of GC cells.

The *ECHDC2* gene is located on human chromosome 1, covering a genomic region of approximately 32kbp. It encodes a protein of about 292 amino acids. Previous research on the specific role of ECHDC2 in lipid metabolism and aerobic glycolysis has been limited. Yao Yang et al. found that ECHDC2 can be used as an independent prognostic indicator of HBV-associated HCC, and the lipid metabolism and other related pathways were significantly enriched in the ECHDC2 group with high expression (Yang et al. 2018).Lindsay J Wheeler et al. used a multi-omics approach to identify ECHDC2 regulation of fatty acid metabolism in ovarian cancer (Wheeler et al. 2019). Our study underscores the potential of targeting ECHDC2 in GC therapy, highlighting its role in regulating energy metabolism by inhibiting glycolysis. This approach seeks to disrupt cancer cells’ energy supply and redirect their metabolism towards oxidative phosphorylation, thereby curbing cancer progression (Venit et al. 2023). Developing ECHDC2-targeted treatments for GC requires careful design to protect healthy cells and avoid toxicity, along with an efficient delivery system to ensure the treatment reaches the tumor. Since ECHDC2 is often less expressed in GC cells, focusing on patients with low ECHDC2 and considering gene therapy to increase its expression may offer new treatment possibilities.

MCCC2 plays a significant role in cellular metabolism, energy production, and tumor development. Tumor cells require a substantial amount of energy and biosynthetic raw materials for rapid growth and division, increasing their dependence on metabolic pathways (Pietrobon 2021). MCCC2 may play a dual role in this process: on one hand, it contributes to providing essential biosynthetic raw materials for tumor cells by participating in the metabolism of branched-chain amino acids; on the other hand, aberrant expression or function of MCCC2 may be associated with the metabolic reprogramming of tumor cells, affecting tumor growth and development (Meierhofer et al. 2016; Liu et al. 2023). He et al. found that MCCC2 can play an oncogenic role in prostate cancer by regulating the GLUD1-P38 MAPK signaling pathway (He et al. 2020). In previous studies, the P38 MAPK pathway was found to promote the expression of membrane transport proteins, such as GLUT1, to facilitate the entry of glucose into the cell (Zhou et al. 2021). In our study, MCCC2 is identified as a downstream target of *ECHDC2* in inhibiting aerobic glycolysis and proliferation of GC cells. Given the extensive role of P38 MAPK signaling and MCCC2, this regulation has profound implications, extending beyond glycolysis to potentially affect other cellular processes, suggests that ECHDC2 may have a broader regulatory role in tumor cell metabolism and survival.

NEDD4 is a E3 ubiquitin protein ligase in eukaryotes. Ubiquitin ligases are key enzymes in the ubiquitination process, responsible for attaching ubiquitin, a small protein molecule, to target proteins. Ubiquitination is a post-translational modification process that attaches ubiquitin to substrate proteins, thereby affecting their stability, localization, activity, or other functions (Damgaard 2021). NEDD4 plays a role in a variety of cellular processes, including protein degradation, signal transduction, endocytosis, and membrane protein regulation. Studies have shown that NEDD4 is dysregulated in various types of cancer, potentially promoting tumor development by affecting cell proliferation, apoptosis, metabolism, migration, and invasion (Mao et al. 2022; Shao et al. 2018). NEDD4 has been conclusively shown to promote the ubiquitination and degradation of P38 MAPK and PKM2, offering critical clues to its role in cell signal transduction (Jiang et al. 2019; Ding et al. 2021). Based on this, our study delves further, revealing the critical function of the ECHDC2-NEDD4-MCCC2 axis in modulating the P38 MAPK signaling pathway and glycolysis in GC cells. This not only broadens our understanding of NEDD4’s roles within the P38 MAPK signaling pathway and glycolysis but also provides valuable insights for exploring new strategies in GC treatment.

Current research on the interactions between enoyl-CoA hydratase/isomerase family members and E3 ubiquitin ligases is relatively limited. Existing studies have primarily focused on the interactions between E3 ubiquitin ligases and enoyl-CoA hydratase/isomerase family members, which facilitate the ubiquitination and degradation of the latter. For instance, Liu L et al. has shown that the E3 ubiquitin ligase TRIM32 can interact with CDYL to promote its ubiquitination and degradation (Liu et al. 2022). Our study builds upon this field by revealing that the interaction between ECHDC2 and the E3 ubiquitin ligase NEDD4 impacts the ubiquitination levels of the substrate MCCC2. This suggests that the interaction between ECHDC2 and NEDD4 could enhance the ubiquitination of MCCC2 by altering NEDD4’s structure or catalytic efficiency. Daniel Horn-Ghetko et al. have shown how SCF-RBR E3-E3 super-assembly enhances ubiquitination levels through promoting interactions and conformational changes among substrates (Horn-Ghetko et al. 2021). Michał Tracz et al. discovered that the E3 ubiquitin ligase SPL2, when bound to lanthanide elements like Ca2 + and La3+, undergoes a conformational change (Tracz et al. 2021). Additionally, such interactions could also recruit cofactors that boost the ligase activity or specificity of NEDD4 towards MCCC2. Emily Yang et al. emphasizes the importance of specific interactions between TRIM25 and R54P on the activity of TRIM25 (Yang et al. 2022). Indranil Paul et al. have shown how the E3 ubiquitin ligase CHIP interacts with various cofactors such as BAG2, BAG3, BAG5, and HSJ1a, regulating its activity through different mechanisms (Paul and Ghosh 2014). The connection between the enoyl-CoA hydratase/isomerase family members and ubiquitination highlights the multifaceted roles these enzymes play in cellular regulation, extending beyond their traditional metabolic functions. This connection encourages further research into how manipulating these enzymes or their interactions with the ubiquitination pathway can exploit the interplay between metabolism and protein regulation to offer new therapeutic approaches for cancer treatment.

## Conclusions

**Fig. 7:**
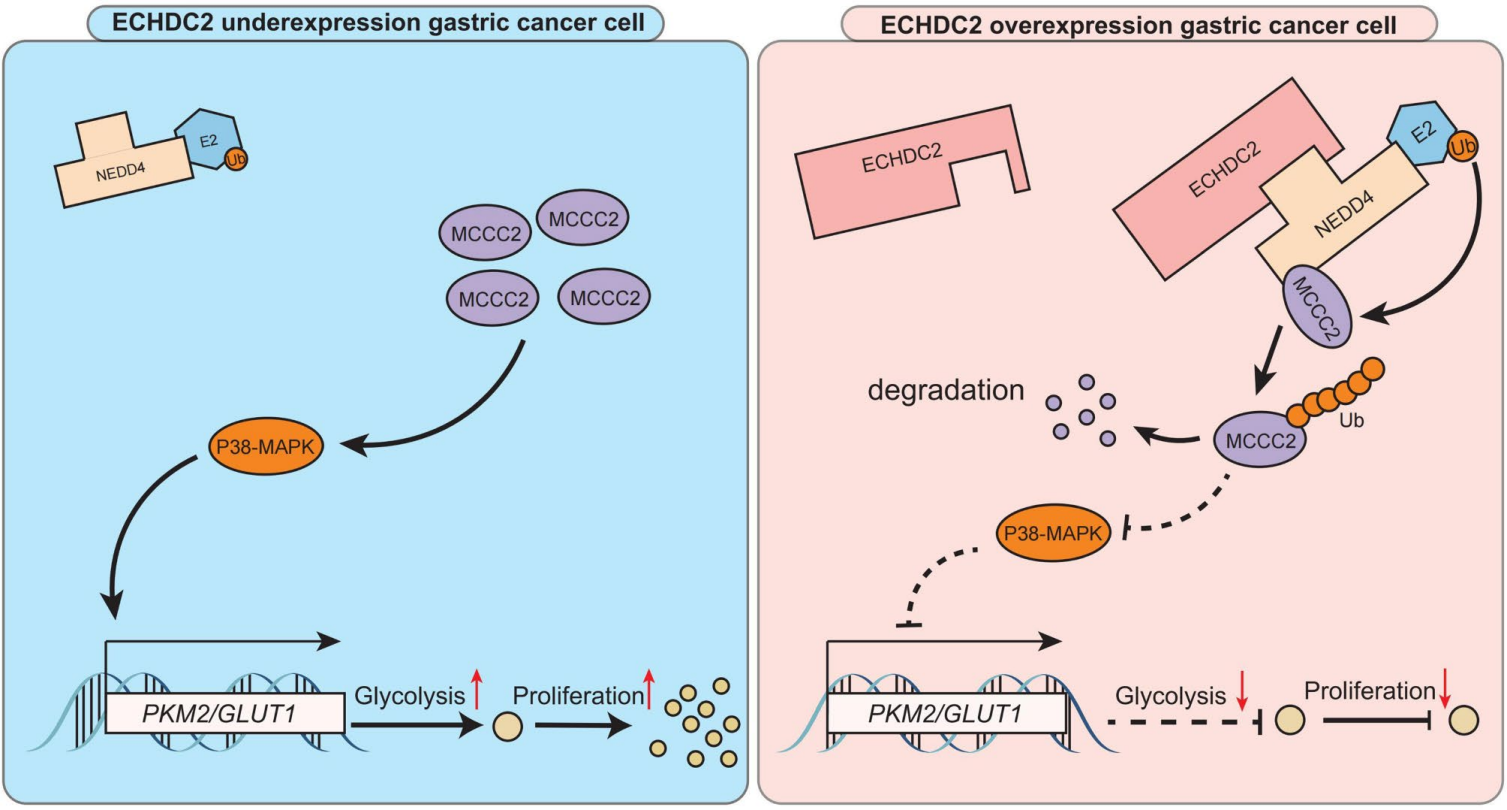
ECHDC2 inhibits the proliferation of GC cells by binding with NEDD4 to degrade MCCC2 and reduce aerobic glycolysis. The schematic of the mechanism of action of ECHDC2 in GC cells. In GC tissues with ECHDC2 overexpression, ECHDC2 binds to NEDD4 to degrade MCCC2. This reduces the activation of P38 MAPK and its downstream targets GLUT1 and PKM2, thereby inhibiting aerobic glycolysis and proliferation in GC cells

We have identified a novel GC suppressor gene, *ECHDC2*, which is downregulated in GC and positively correlated with poor prognosis. Mechanistically, ECHDC2 mediates the ubiquitination and degradation of MCCC2 by NEDD4, which in turn inhibits the P38 MAPK signaling pathway. This suppression leads to reduced expression of downstream genes *PKM2* and *GLUT1*, thereby inhibiting aerobic glycolysis and proliferation in GC cells (Fig. 7). This discovery provides a new perspective for understanding the regulatory mechanisms of aerobic glycolysis and the pathogenesis of GC. It also highlights the significance of ECHDC2 as a potential therapeutic target for GC.

## Methods

### Clinical and tissue samples

Human GC samples (*n* = 136) and matched normal tissues collected during surgical resection at the Affiliated Hospital of Nantong University from 2014 to 2016. Clinicopathologic information is shown in Table 1. Between 2020 and 2021, another set of 20 fresh GC tissues and adjacent normal tissues were collected from the same source for qRT-PCR and western blotting. All patients were pathologically diagnosed with GC, and none of these patients received chemotherapy or radiotherapy before surgery. The study was approved by the Ethics Committee of the Affiliated Hospital of Nantong University (2023-K076-01), and all patients provided informed consent.

### Cell culture and reagents

GC cell lines MKN-45, HGC-27, MGC-803 and normal gastric mucosa cells lines GES1 were purchased from GeneChem (Shanghai, China). GC cell lines NCI-N87 was purchased from Procell (Wuhan, China). GC cells were cultured in 1640 medium containing 10% fetal bovine serum, 1% penicillin (100 U/ml), and 1% streptomycin (100 µg/ml) in an incubator containing 5% CO_2_ at 37 °C. Cycloheximide (CHX), MG132 and Chloroquine (CQ) were purchased from MedChemExpress (Shanghai, China).

### Cell transfection and establishment of stable transfected cell lines

ECHDC2, P38 MAPK and MCCC2 overexpression plasmid and NEDD4 siRNA were purchased from Genepharma (Shanghai, China). Cell transfection was performed on six-well plates using jetPRIME reagents, purchased from Polyplus (Strasbourg, France). After plasmid transfection, cells were selected with G418 to establish stable transfected cell lines. G418 was purchased from TransGen Biotech (Beijing, China).

### RNA isolation and quantitative real-time polymerase chain reaction (qRT-PCR)

Total RNA was extracted from GC cells using TRIzol reagent (Invitrogen, USA). qRT-PCR was performed following a previously described method (Zang et al. 2022). The primers are shown in Table 2.

**Table 2:** Primer sequence

ECHDC2	Forward: CTCTTCAGAAGTGGAGTGAAGG Reverse: GGTCTCAATCAGTCCCATGAC
MCCC2	Forward: GAAAGTCTGGAGTAAGTGACCA Reverse: CATCAGCAGGAAATAAAGGCTC
GLUT1	Forward: CTCCGGTATCGTCAACACGG Reverse: AAGCCAAAGATGGCCACGAT
PKM2	Forward: TCGCATGCAGCACCTGATT Reverse: CCTCGAATAGCTGCAAGTGGTA
SDHA	Forward: AGGCTTGCGAGCTGCATTTG Reverse: AGCCCTTCACGGTGTCGTAG
G6PD	Forward: AAACGGTCGTACACTTCGGG Reverse: GGTAGTGGTCGATGCGGTAG
GAPDH	Forward: TCGACCACAACTGCTTAGC Reverse: GGCATGGACTGTGGTCATGAG

### Western blotting assay

Western blotting assay were performed as described previously (Zang et al. 2022). The antibodies used were as follows: anti-MCCC2 (12117-1-AP, Proteintech, China); anti-ECHDC2 (bs-13049R, Bioss, China); anti-GLUT1 (21829-1-AP, Proteintech, China); anti-PKM2 (15822-1-AP, Proteintech, China); anti-SDHA (14865-1-AP, Proteintech, China); anti-G6PD (ab993, Abcam, UK); anti-c-Myc (10828-1-AP, Proteintech, China); anti-p-AKT (66444-1-lg, Proteintech, China); anti-P38 MAPK (14064-1-AP, Proteintech, China); anti-ubiquitin (10201-2-AP, Proteintech, China); anti-NEDD4 (21698-1-AP, Proteintech, China); anti-ITCH (20920-1-AP, Proteintech, China); anti-CBL (25818-1-AP, Proteintech, China); anti-GAPDH (10494-1-AP, Proteintech, China).

### Glucose and lactic acid assay

Logarithmically grown cells (1 × 10^6^) were taken and inoculated in six-well plates, the medium was collected after 12 h of replacement and filtered through a 10-kD spin column. Glucose and lactic acid were measured using glucose assay kit (Bioss, China) and lactic acid assay kit (Njjcbio, China) respectively. Glucose uptake was determined by subtracting the final medium glucose concentration from the initial medium glucose concentration in the medium. Data were obtained from three independent triplicates and normalized to cell number.

### Colony formation assay, CCK-8 and EDU assay

Colony formation assay and CCK-8 were performed as described previously (Zang et al. 2022). In the colony formation assay, cells in the logarithmic growth phase were digested, resuspended in complete medium, and repeatedly pipetted to disperse into a single cell suspension. Cell counts were conducted, and cells were seeded in 6-well plates at a density of 1000 cells per well, with a total culture volume of 4 ml and 6 replicate wells per group. The plates were then incubated at 37 °C in a 5% CO2 incubator. The medium was changed regularly, and the culture was continued for 2 to 3 weeks. Once colonies visible to the naked eye appeared in the plates, the culture was terminated. The supernatant was removed, and the cells were washed twice with PBS buffer, fixed with 4% paraformaldehyde for 30 min, washed again twice with PBS buffer, stained with Crystal Violet Staining Solution at room temperature for 15 min, washed again twice with PBS buffer, and finally photographed for observation. Clone counts were performed using ImageJ to calculate cloning efficiency. For the CCK-8 assay, cells were seeded in 96-well plates at a density of 3000 cells per well, with approximately 100 µl of cell suspension per well and 6 replicate wells per group. Sterile PBS was used to fill the peripheral wells, and the cell plate was gently shaken to ensure even distribution of cells, then incubated at 37 °C in a 5% CO2 incubator for 0 h, 24 h, 48 h, and 72 h. In a light-protected environment, 10 µl of CCK-8 solution was gently added to each well, shaken gently to ensure even mixing and to avoid bubble formation, and incubated for 1 h at 37 °C. Absorbance at 450 nm was measured using a microplate reader. The EDU assay was performed using the BeyoClick™ EdU-555 Cell Proliferation Assay Kit (Beyotime, Shanghai, China) according to the manufacturer’s instructions. Images were captured using a Leica Thunder fully automated inverted microscope.

### Immunofluorescence (IF) assay

IF assays were performed as previously described (Zhu et al. 2021). Briefly, treated cells were washed, fixed, permeabilized, and blocking. Subsequently, the cells were co-incubated overnight with an anti-NEDD4 (21698-1-AP, Proteintech, China) antibody at 4 °C, followed by three washes with PBS. Next, the cells were incubated for one hour with ABflo 488-conjugated Goat Anti-Rabbit IgG (H + L) (AS053, ABclonal, China), then washed three times with PBS. The cells were then incubated overnight with the anti-ECHDC2 (bs-13049R, Bioss, China) antibody at 4 °C, followed by washing with PBS. Afterward, the cells were incubated for one hour with ABflo 555-conjugated Goat Anti-Rabbit IgG (H + L) (AS058, ABclonal, China), then washed three times with PBS, followed by overnight incubation with the anti-MCCC2 (12117-1-AP, Proteintech, China) antibody at 4 °C, and subsequent PBS washing. Finally, the cells were incubated for one hour with ABflo 647-conjugated Goat Anti-Rabbit IgG (H + L) (AS060, ABclonal, China), and stained for 15 min with DAPI (Cell Signaling Technology, USA). Imaging of stained cells was captured using a Zeiss LSM 900 confocal microscope.

### Tissue microarray (TMA), immunohistochemistry (IHC) and fluorescence multiplex immunohistochemistry (mIHC)

GC tissue specimens and matched, normal, tumor-adjacent tissues (*n* = 136) were prepared and used for TMAs. We used Tissue Microarray System (Quick-Ray, UT06, UNITMA, Korea) in the department of clinical pathology, Nantong University Hospital, Jiangsu, China. Core tissue biopsies (2 mm in diameter) were taken from individual paraffin-embedded sections and arranged in recipient paraffin blocks. TMA specimens were cut into 4-µm sections and placed on super frost-charged glass microscope slides. IHC was performed as previously described (Zhu et al. 2021). The primary antibody used were as follows: anti-ECHDC2 (bs-13049R, Bioss, China); anti-GLUT1 (21829-1-AP, Proteintech, China); anti-PKM2 (15822-1-AP, Proteintech, China); anti-Ki67 (ab15580, Abcam, UK). Staining intensity was manually scored by two independent experienced pathologists and a third pathologist was invited to reassess when large discrepancies occurred. Staining intensity was scored: 3 (strongly positive), 2 (moderately positive), 1 (weakly positive) and 0 (negative). Scoring was then based on the positivity rate: 4 (> 75%), 3 (> 50-75%), 2 (> 25-50%), 1 (5-25%), and 0 (< 5%). The final score was the positivity score multiplied by the staining intensity score. The scores were categorized according to the score as ECHDC2 high expression (score ≥ 3) and ECHDC2 low expression (score ≤ 2) (Zang et al. 2022). We included both negative controls (normal liver tissue without primary antibody) and positive controls (normal liver tissue) to support the validity of our staining outcomes. mIHC was conducted using the mIHC staining kit (absin, Shanghai, China) following the manufacturer’s instructions. The following primary antibodies were employed: anti-ECHDC2, anti-NEDD4, and anti-MCCC2.

### Coimmunoprecipitation (Co-IP)

Co-IP was performed as previously described (Liu et al. 2020a, b). Total cell lysates were incubated with primary antibody at 4 °C overnight. Protein A + G agarose (Bioworld Technology, St. Louis Park, MN, USA) was added and then further incubated at lower 4 °C. Beads were collected by centrifugation after rinsing with PBS. Finally, samples were detected by western blotting.

### Animal experiment

Ten 4-week-old male nude mice purchased from the Model Animal Research Center of Nantong University (Nantong, China) were randomly divided into two groups: the control group and the overexpression ECHDC2 group, with five mice in each group. 200 µl cell suspension (2 × 10^6^ cells) was injected into the right axillary subcutis of each nude mouse. The growth of the nude mice was regularly observed, and the volume and weight of the subcutaneous tumors were measured every three days. 22 days later, the nude mice were executed, and the tumors were peeled off, weighed, photographed, recorded, and further plotted on the growth curve of the subcutaneous tumors. All animal experiments were approved by the Laboratory Animal Ethics Committee of Nantong University.

### Protein-protein docking

PDB structures of ECHDC2, NEDD4, and MCCC2 were obtained from UniProt (https://www.uniprot.org/). Docking was performed using GRAMM (https://gramm.compbio.ku.edu/gramm). PDBePISA (https://www.ebi.ac.uk/msd-srv/prot_int/pistart.html) was employed for the analysis of the docking results, and the docking models with the lowest free energy were represented using PyMOL (https://pymol.org/).

### Data acquisition and processing

TCGA-STAD gene expression data were downloaded from The Cancer Genome Atlas (TCGA, https://portal.gdc.cancer.gov/) database. GSE27342, GSE54129 datasets were downloaded from the Gene Expression Omnibus (GEO, https://www.ncbi.nlm.nih.gov/geo/) database. The data underwent standardized preprocessing and were transformed using the log2 (value + 1) formula within the R programming environment. First, extract the expression levels of each gene within the Enoyl-CoA hydratase/isomerase family from the organized data, categorizing them into tumor and normal groups. Subsequently, each gene’s expression data were subjected to tests for normality and homogeneity of variance. The TCGA-STAD dataset was analyzed using paired sample T-tests, while the GSE27342 and GSE54129 datasets were examined using the Mann-Whitney U test. *p* < 0.05 was considered the criterion for statistically significant differences.

### Statistical analysis

Data were analyzed using GraphPad Prism 8.0 and the R programming language (version 4.2.3). Measurement data with normal distribution were expressed as mean ± SD and statistically analyzed with two-sample t test. Non-normal distribution measurement data were expressed as median (range) and analyzed by Mann-Whitney U test. The count data were expressed as the number of cases (percentage) and analyzed by chi-square test. For groups of three or more that conform to a normal distribution, one-way ANOVA is utilized, while for those that do not follow a normal distribution, the Kruskal–Wallis test is employed. Survival rates of GC patients were calculated by Log-rank analysis and Kaplan-Meier, and prognostic factors were assessed using COX regression model. All statistical analyses were two-sided, and *P* < 0.05 was used to define statistical significance. All experiments were conducted more than three times.

### Electronic supplementary material

Below is the link to the electronic supplementary material.

Supplementary Material 1

Supplementary Material 2

## Data Availability

The data that support the findings of this study are available on request from the corresponding author.
