# Supplementary material for: ECHDC2 inhibits the proliferation of gastric cancer cells by binding with NEDD4 to degrade MCCC2 and reduce aerobic glycolysis
Source: Mol Med. 2024 May 23;30:69. doi: 10.1186/s10020-024-00832-9 (PMC11118108; doi:10.1186/s10020-024-00832-9)
Supplement: Supplementary file 1 — Supplementary Material 1 [file 10020_2024_832_MOESM1_ESM.docx]

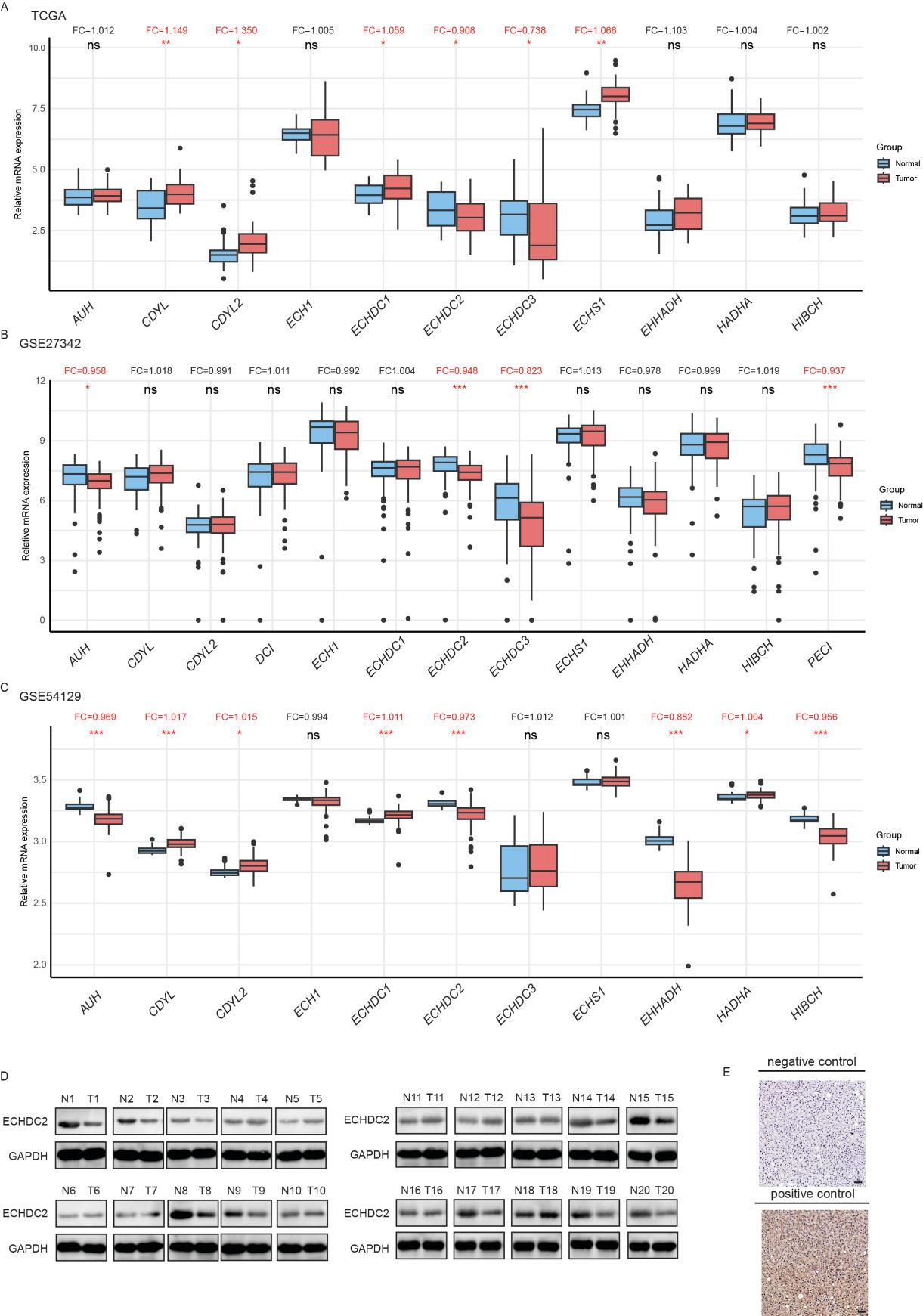


**Figure S1. ECHDC2 is downregulated in GC tissues.**

**(A-C)** Box plot of Enoyl-CoA hydratase/isomerase family genes expression levels in three datasets: TCGA-STAD, GSE27432 and GSE54129 **(D)** Western blotting was utilized to detect the expression of ECHDC2. **(E)** TMA IHC negative control (normal liver tissue without primary antibody) and TMA IHC positive control (normal liver tissue). Scale bar, 50μm. ns. P>0.05, * P<0.05, ** P<0.01, *** P<0.001.


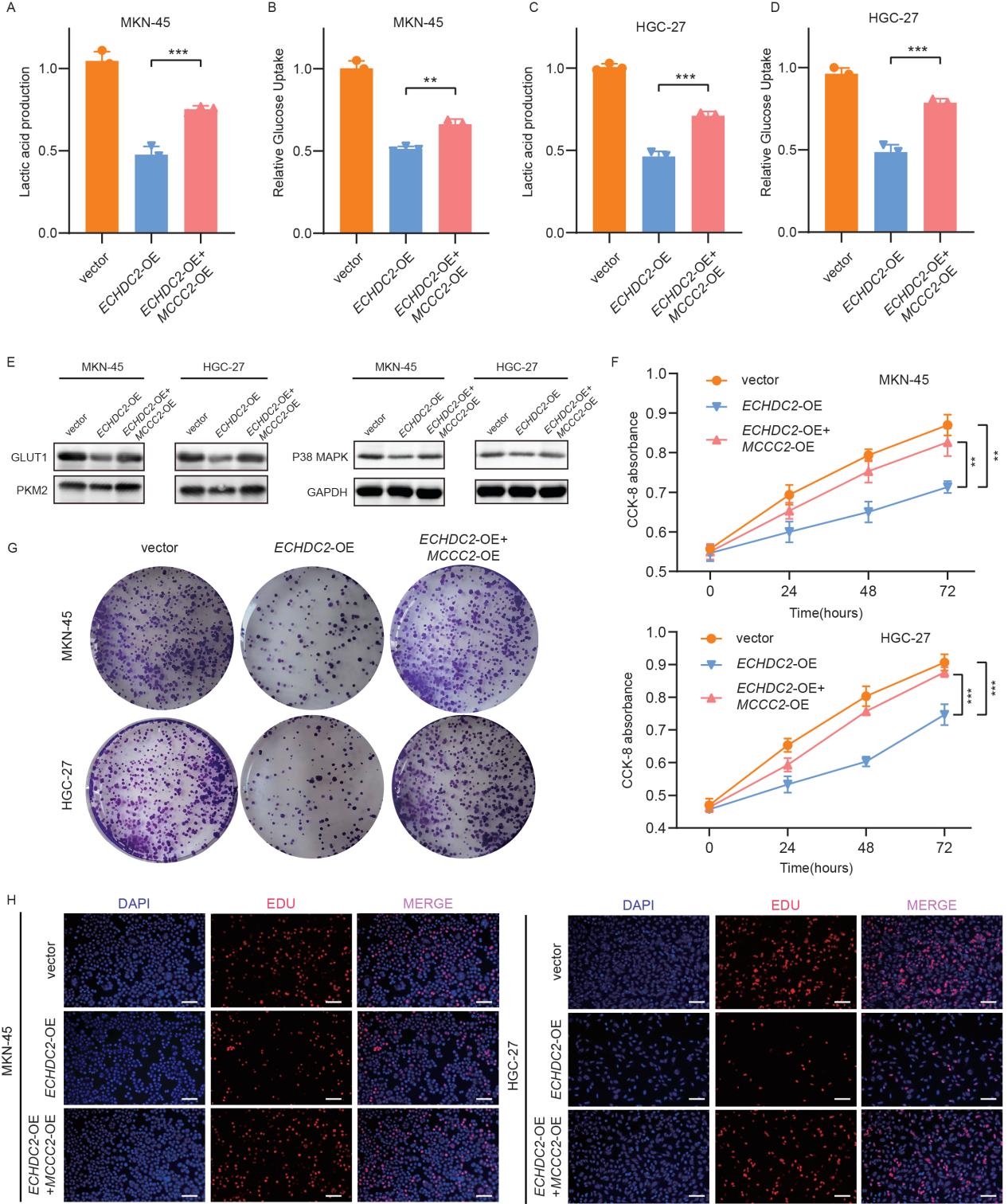


**Figure S2. MCCC2 mediates the inhibition of aerobic glycolysis and cell proliferation by ECHDC2.**

**(A-D)** Glucose uptake rate and lactic acid production rate of GC cells were measured after ECHDC2 overexpression and/or MCCC2 overexpression. **(E)** Western blotting was utilized to detect the expression of GLUT1, PKM2 and P38 MAPK after ECHDC2 overexpression and/or MCCC2 overexpression. **(F-H)** The cell viability and cell proliferation capacity of GC cells were assessed following ECHDC2 overexpression and/or MCCC2 overexpression by CCK-8 assay, colony formation assay and EDU assay. Scale bar, 20μm. **P<0.01, *** P<0.001.
