# Supplementary figures and images for: ECHDC2 inhibits the proliferation of gastric cancer cells by binding with NEDD4 to degrade MCCC2 and reduce aerobic glycolysis
Source: Mol Med. 2024 May 23;30:69. doi: 10.1186/s10020-024-00832-9 (PMC11118108; doi:10.1186/s10020-024-00832-9)

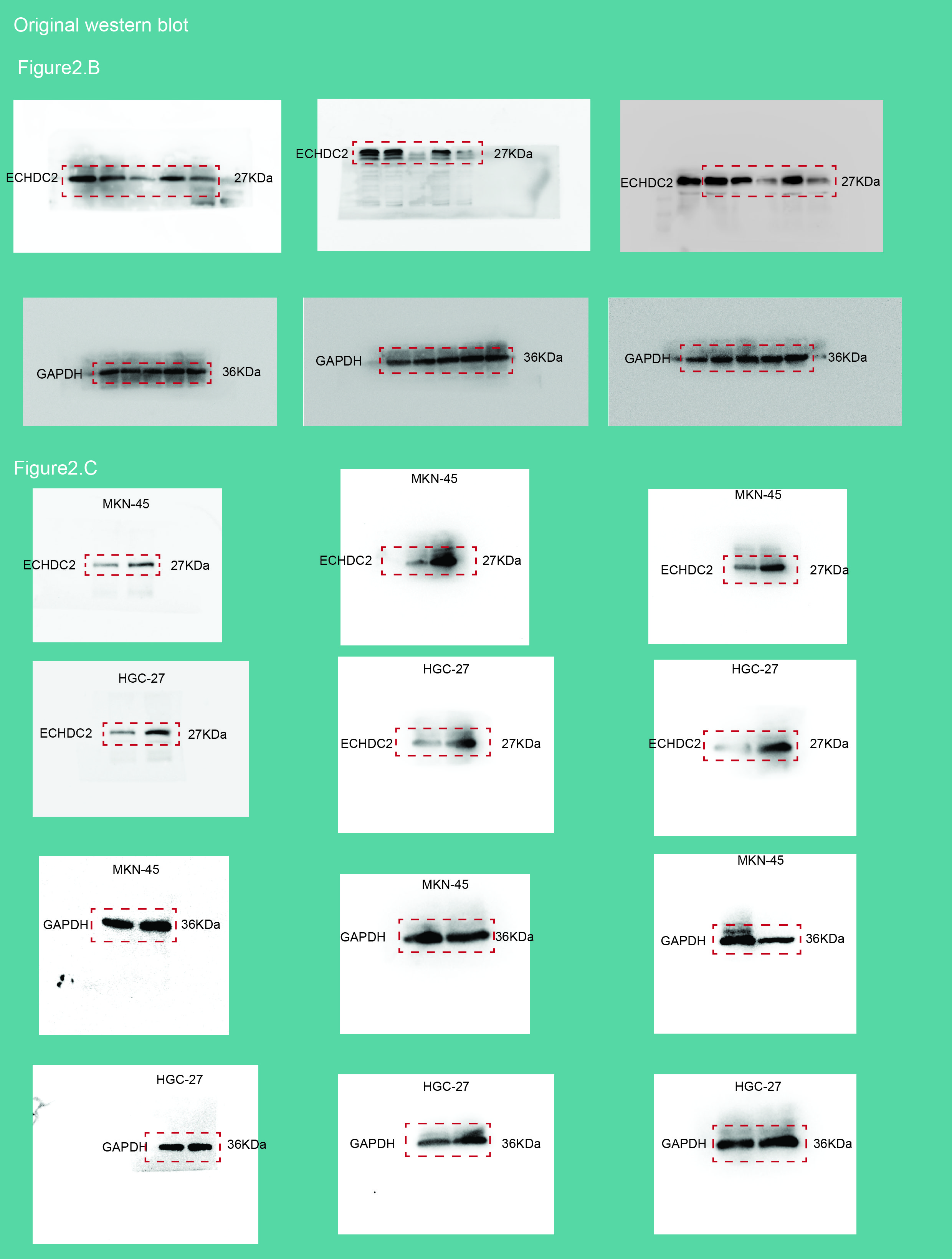

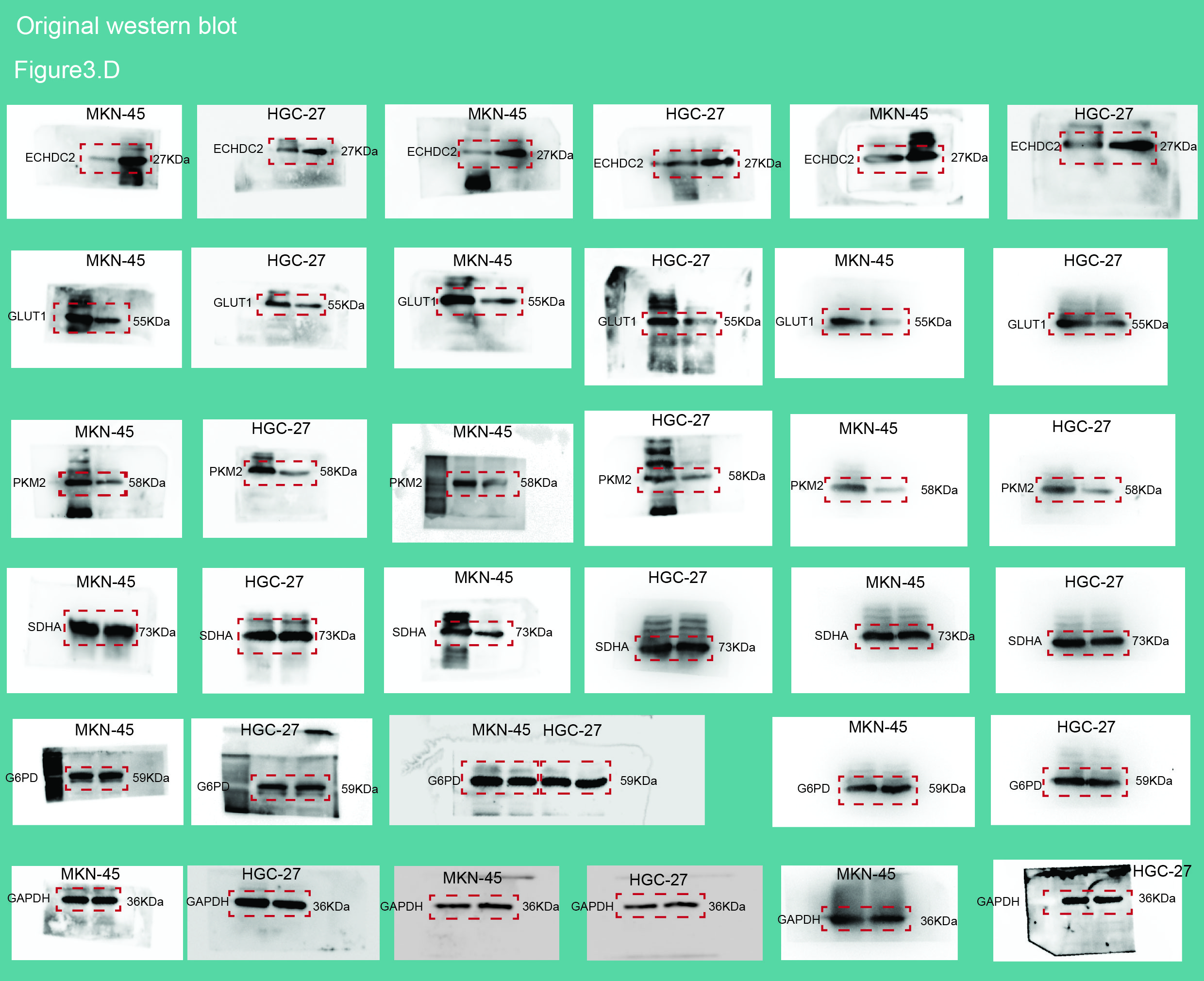

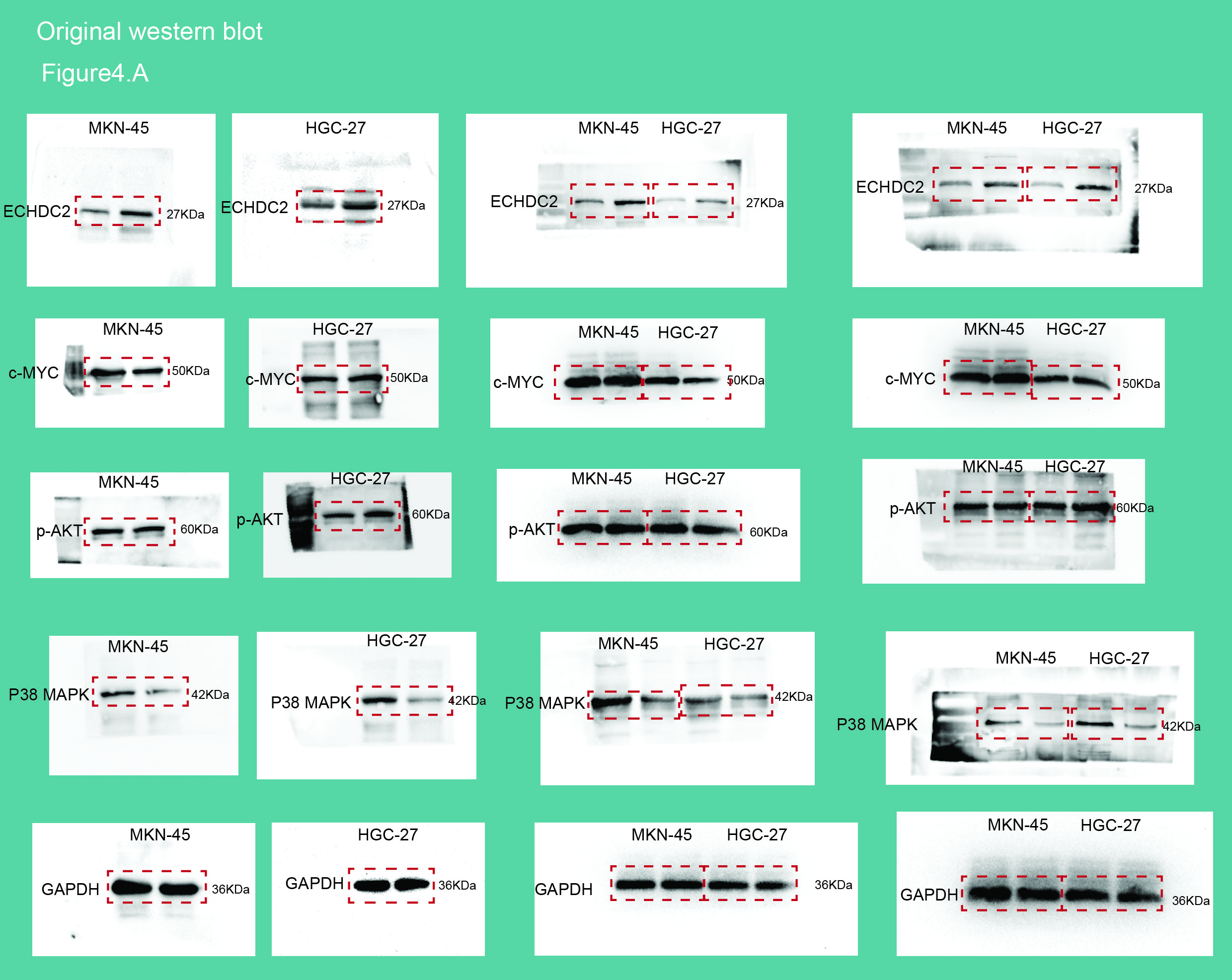

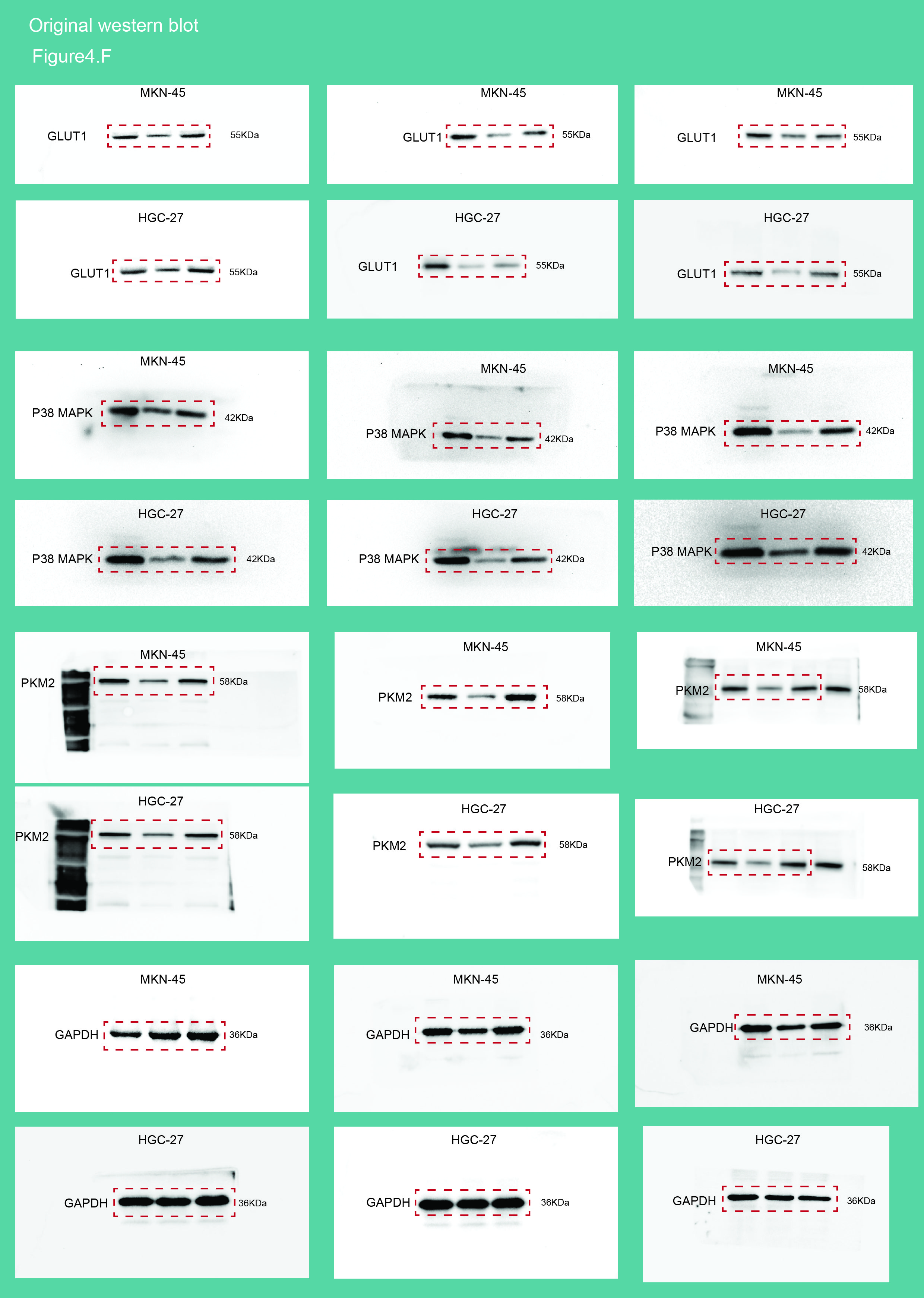

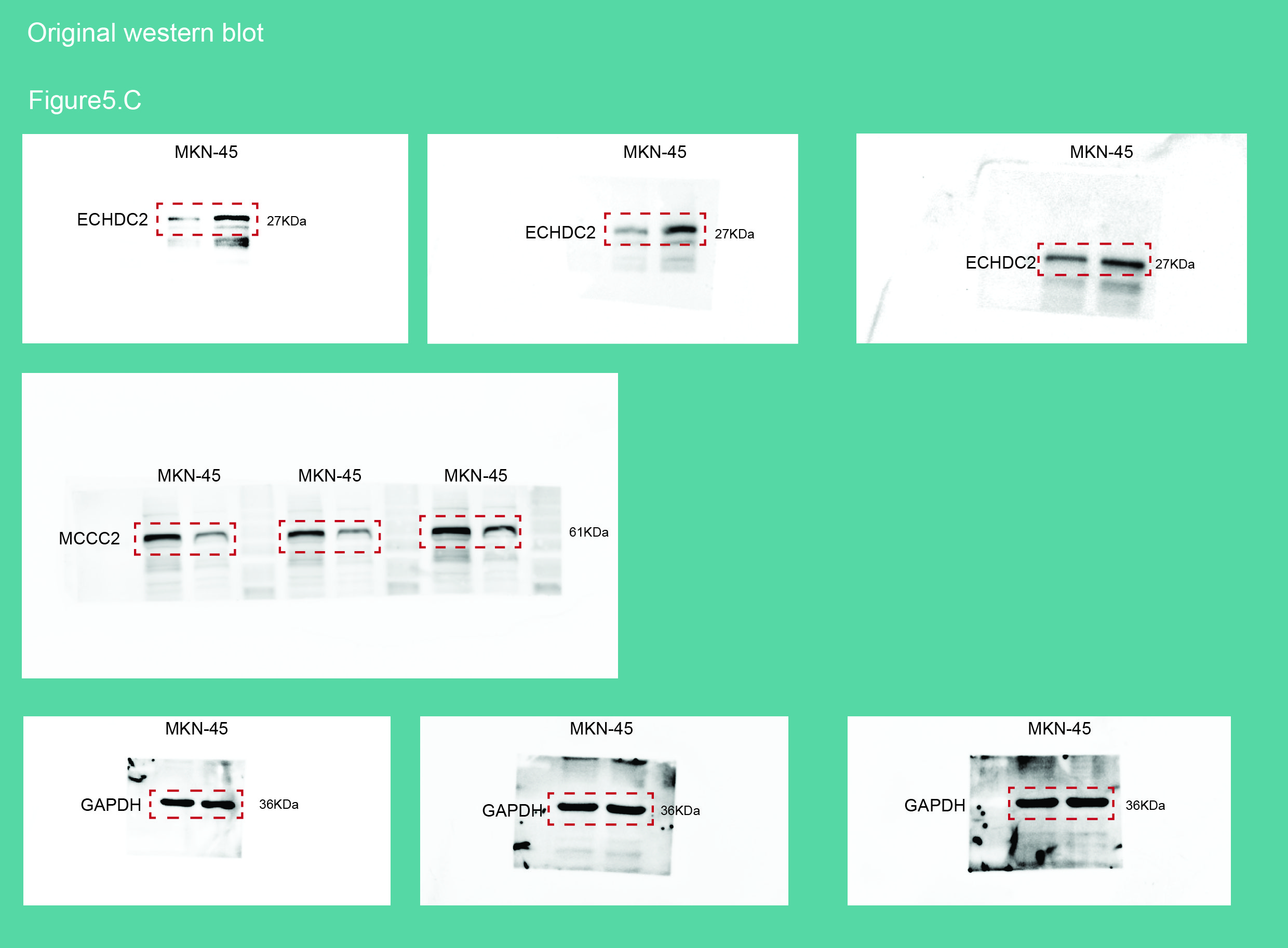

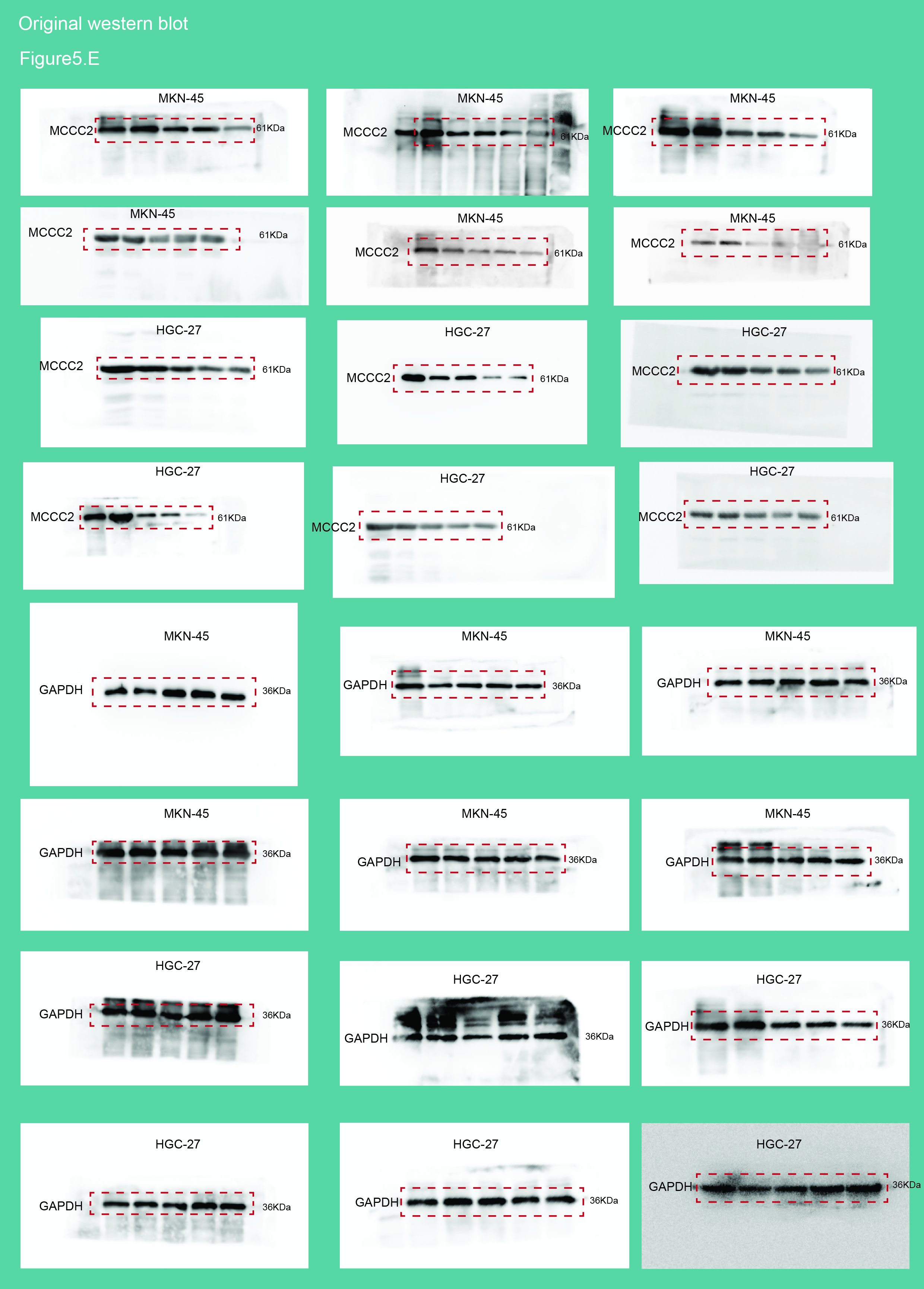

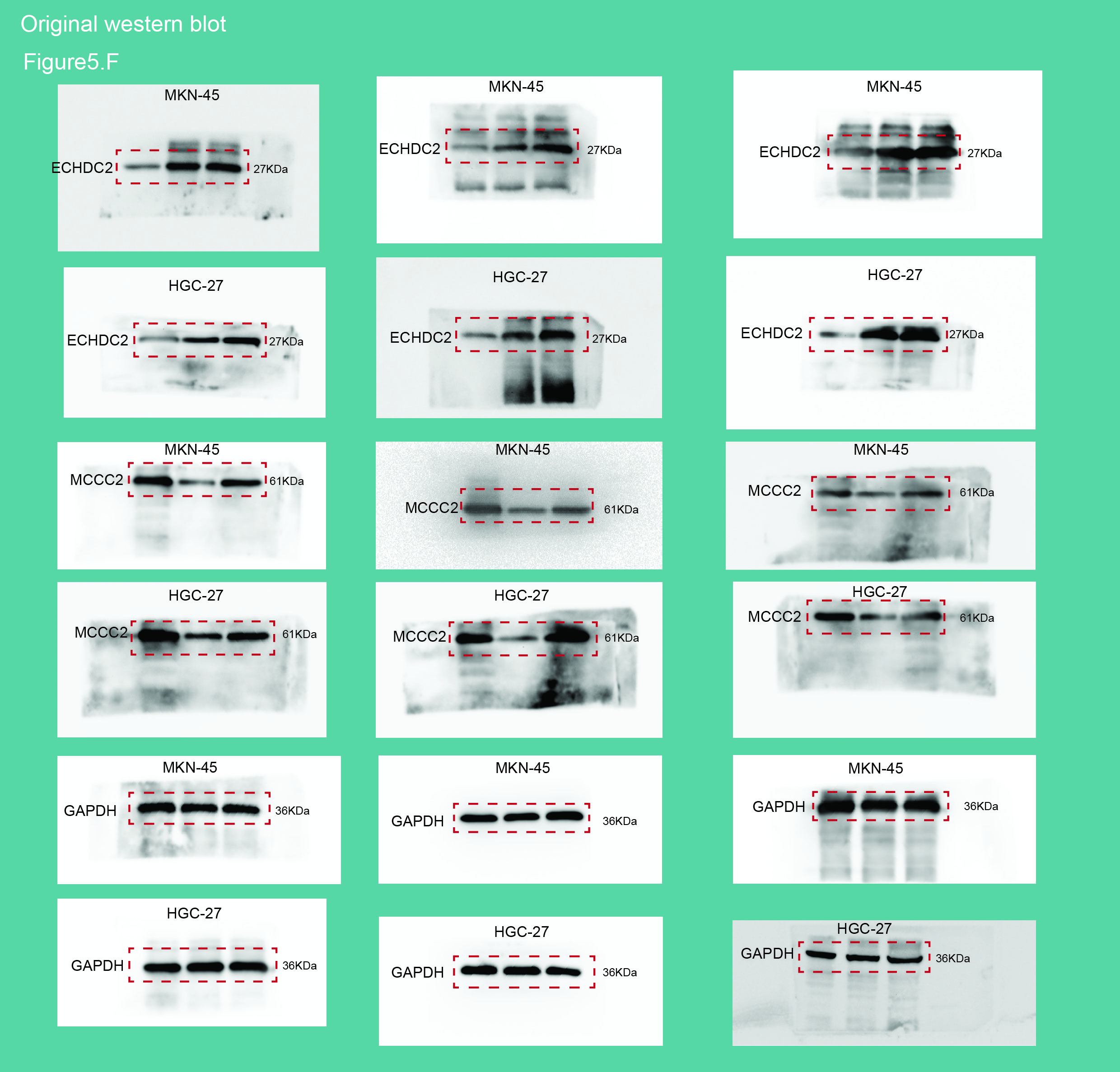

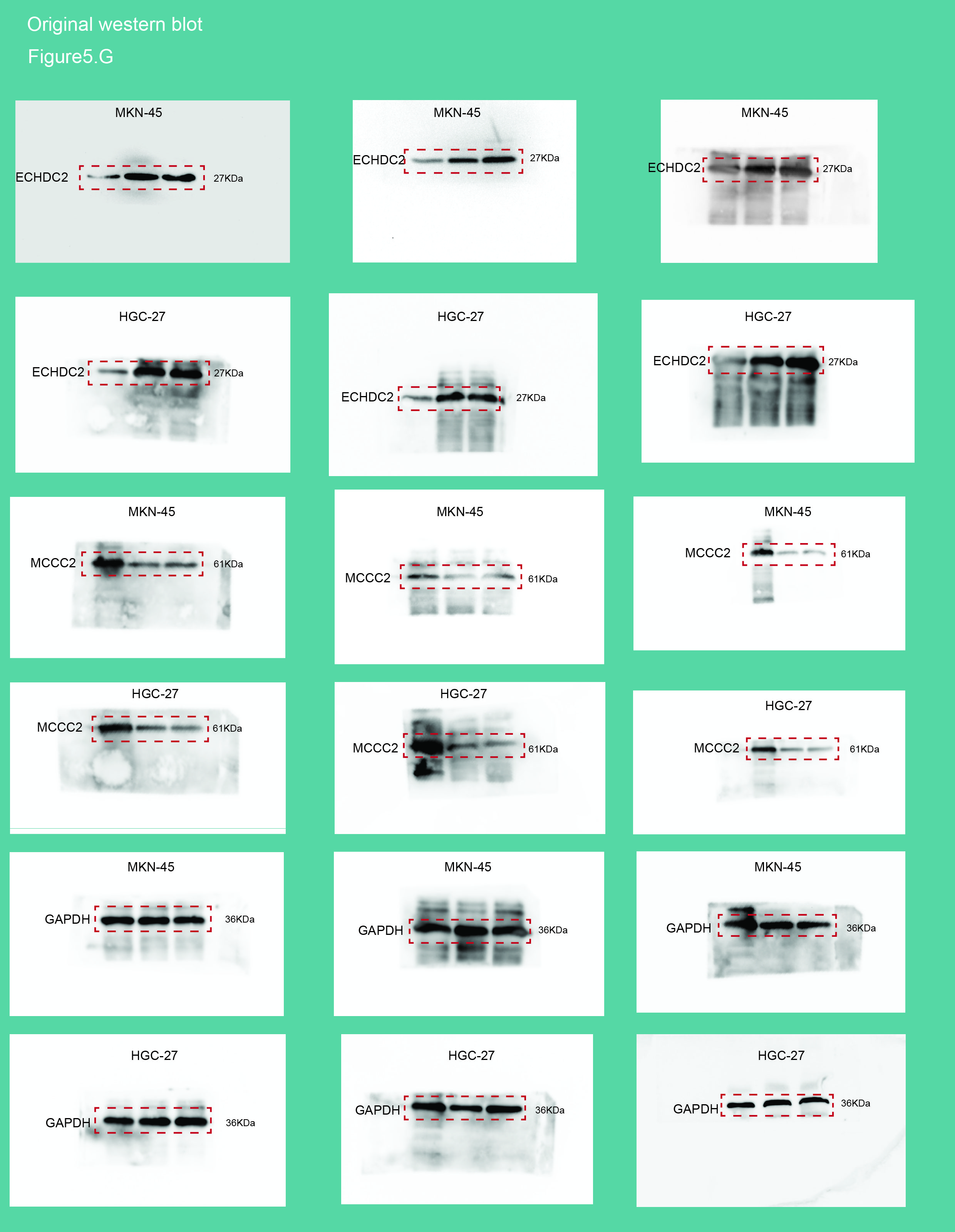

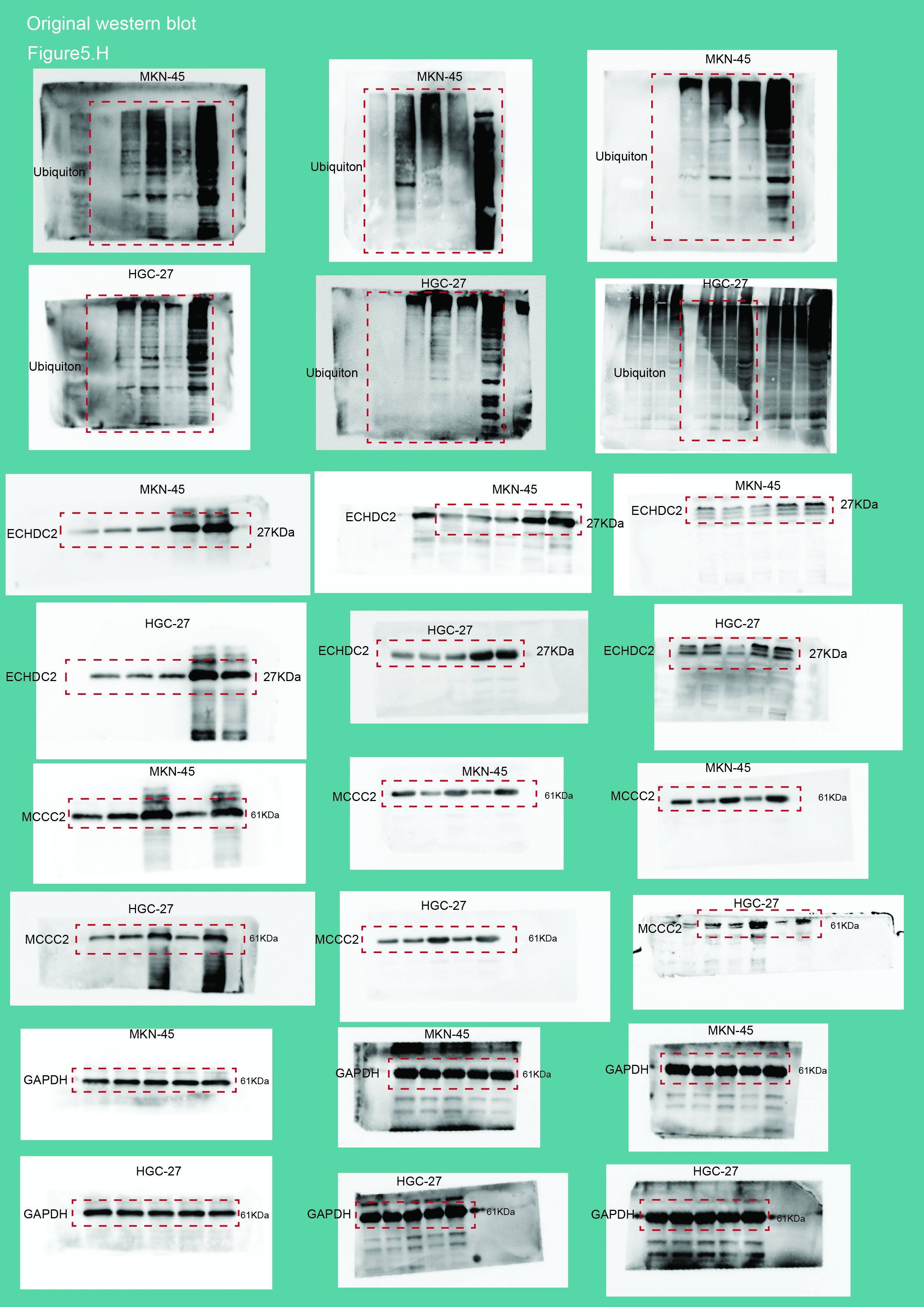

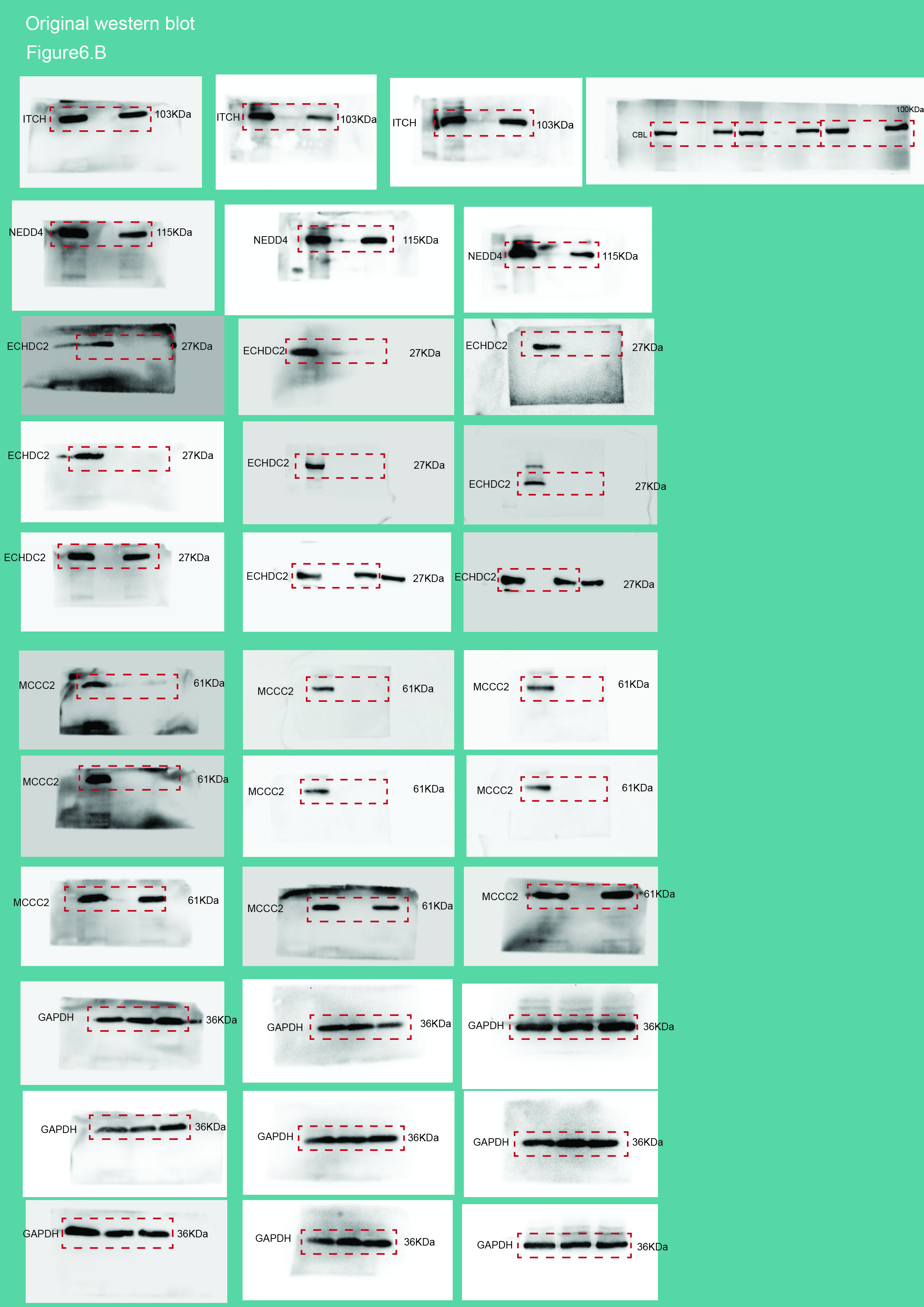

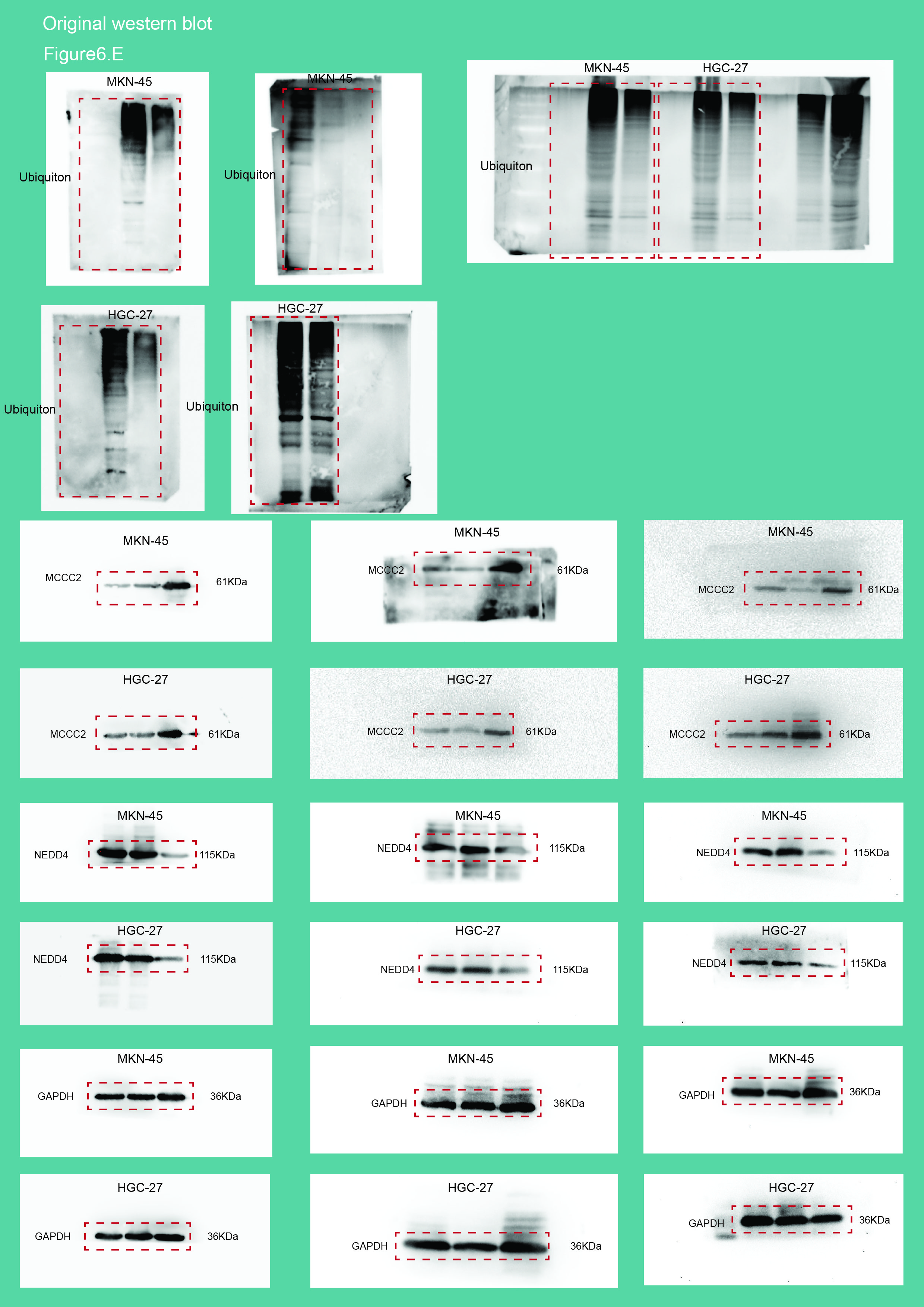

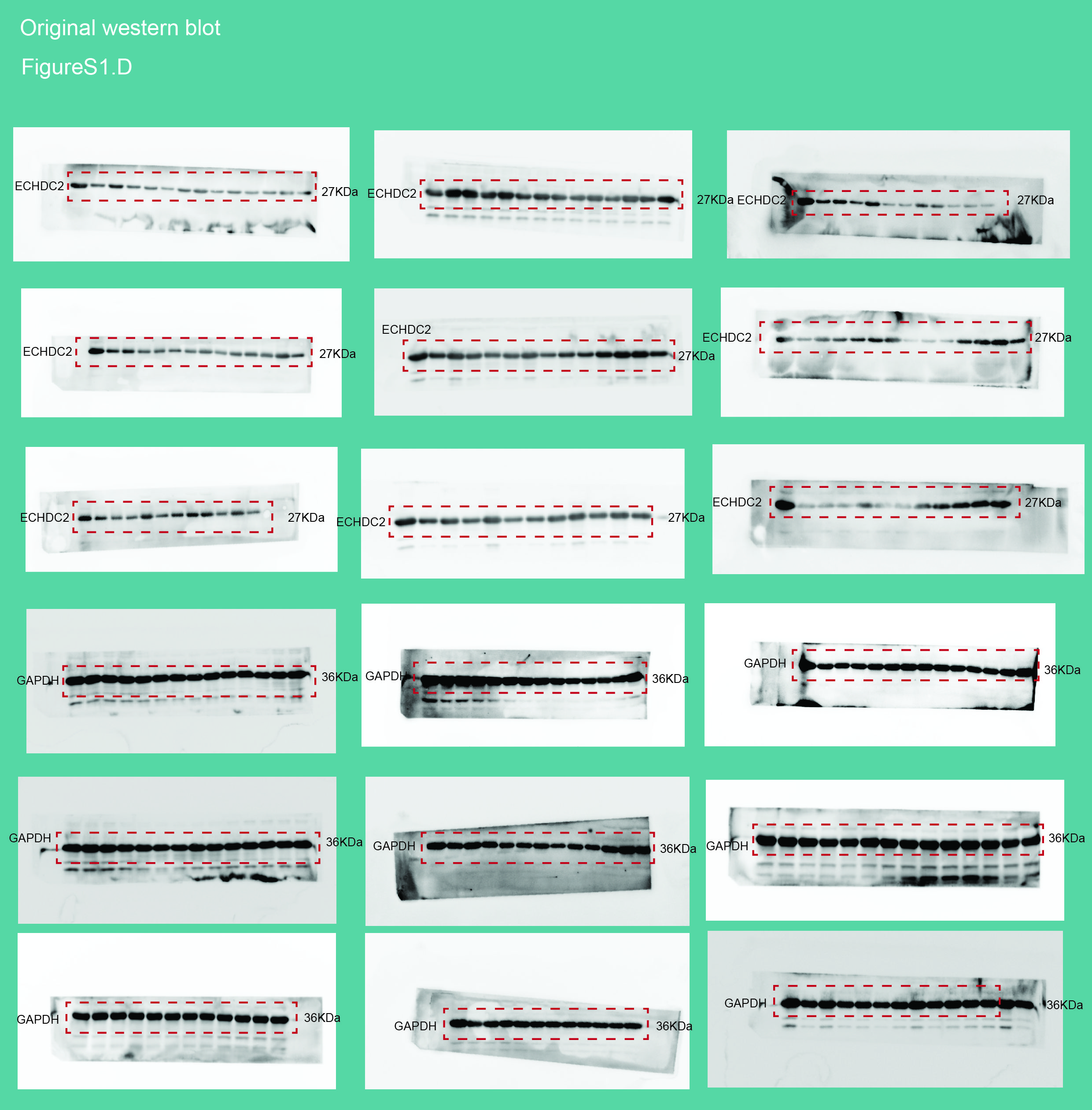

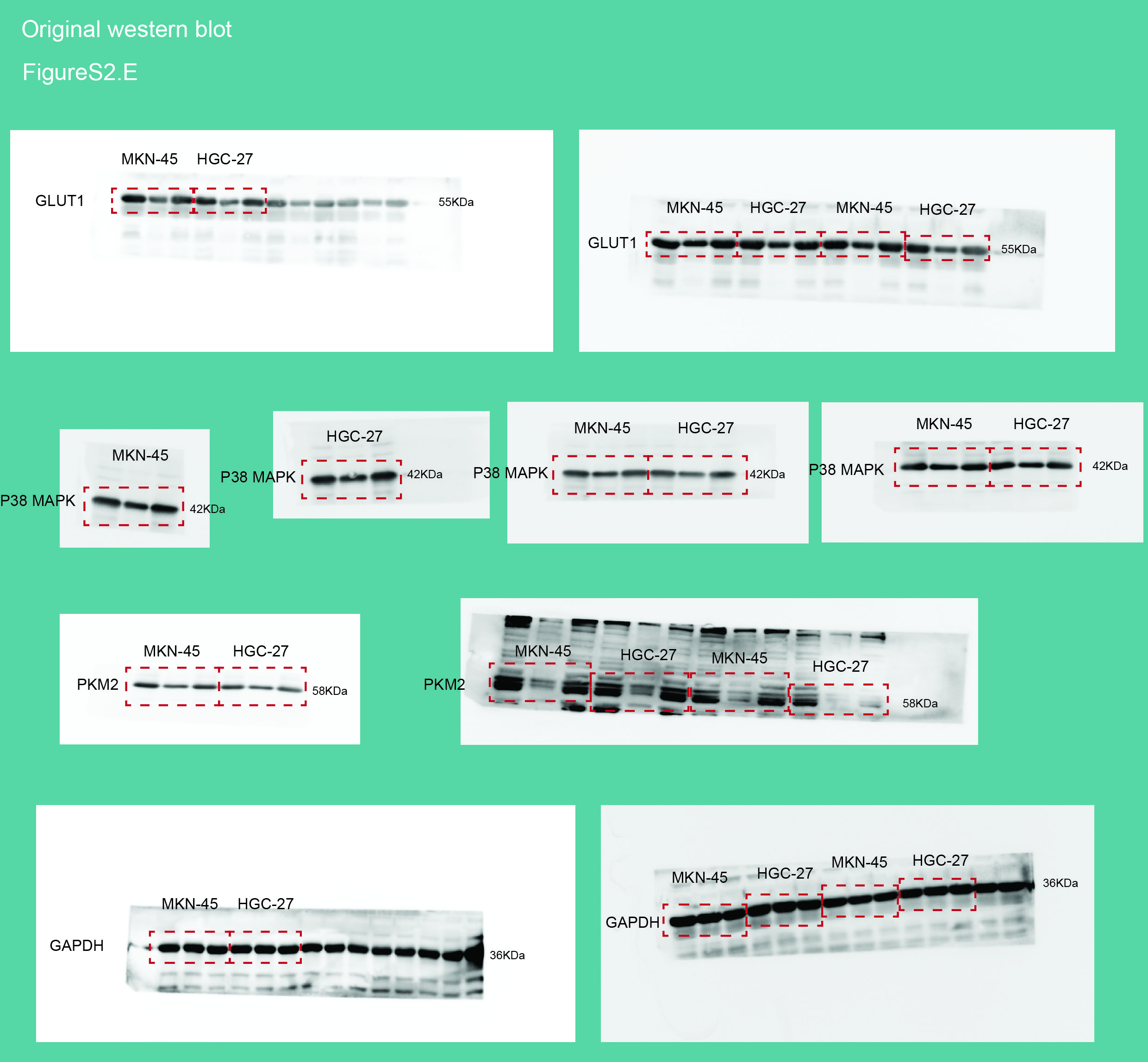

Supplement: Supplementary file 2 — Supplementary Material 2 [file 10020_2024_832_MOESM2_ESM.docx]
